# Phylogeography and evolutionary history of the *Crocidura olivieri* complex (Mammalia, Soricomorpha): from a forest origin to broad ecological expansion across Africa

**DOI:** 10.1186/s12862-015-0344-y

**Published:** 2015-04-23

**Authors:** François Jacquet, Christiane Denys, Erik Verheyen, Josef Bryja, Rainer Hutterer, Julian C Kerbis Peterhans, William T Stanley, Steven M Goodman, Arnaud Couloux, Marc Colyn, Violaine Nicolas

**Affiliations:** Institut de Systématique, Évolution, Biodiversité, ISYEB UMR 7205 - CNRS, MNHN, UPMC, EPHE, Muséum National d’Histoire Naturelle, Sorbonne Universités, 57 rue Cuvier, CP 51, 75005 Paris, France; Royal Belgian Institute of Natural Sciences, Operational Direction Taxonomy and Phylogeny, Molecular Laboratory, Vautierstraat 29, 1000 Brussels, Belgium; Biology Department, University of Antwerpen, Evolutionary Ecology Group, Groenenborgerlaan 171, 2020 Antwerpen, Belgium; Institute of Vertebrate Biology ASCR, Academy of Sciences of the Czech Republic, Květná 8, 603 65 Brno, Czech Republic; Department of Botany and Zoology, Faculty of Science, Masaryk University, Kotlářská 2, 611 37 Brno, Czech Republic; Zoologisches Forschungmuseum Alexander Koenig, Adenauerallee 160, D-53113 Bonn, Germany; College of Professional Studies, Roosevelt University, 430 S Michigan Avenue, Chicago, IL 60605 USA; Field Museum of Natural History, 1400 South Lake Shore Drive, Chicago, IL 60605 USA; Association Vahatra, BP 3972, Antananarivo, 101 Madagascar; Génoscope, Centre National de Séquençage, 2 rue Gaston Crémieux, CP5706, 91057 Evry Cedex, France; Université de Rennes 1, CNRS, UMR 6553 Ecobio, Station Biologique, 35380 Paimpont, France

**Keywords:** *Crocidura olivieri*, Diversification, Forest refuge, Molecular dating, Phylogeography, Pleistocene climate changes, Riverine barrier, Soricidae, Systematics

## Abstract

**Background:**

This study aims to reconstruct the evolutionary history of African shrews referred to the *Crocidura olivieri* complex. We tested the respective role of forest retraction/expansion during the Pleistocene, rivers (allopatric models), ecological gradients (parapatric model) and anthropogenic factors in explaining the distribution and diversification within this species complex. We sequenced three mitochondrial and four nuclear markers from 565 specimens encompassing the known distribution of the complex, i.e. from Morocco to Egypt and south to Mozambique. We used Bayesian phylogenetic inference, genetic structure analyses and divergence time estimates to assess the phylogenetic relationships and evolutionary history of these animals.

**Results:**

The *C. olivieri* complex (currently composed of *C. olivieri*, *C. fulvastra*, *C. viaria* and *C. goliath*) can be segregated into eight principal geographical clades, most exhibiting parapatric distributions. A decrease in genetic diversity was observed between central and western African clades and a marked signal of population expansion was detected for a broadly distributed clade occurring across central and eastern Africa and portions of Egypt (clade IV). The main cladogenesis events occurred within the complex between 1.37 and 0.48 Ma. *Crocidura olivieri* sensu stricto appears polyphyletic and C. *viaria* and C. *fulvastra* were not found to be monophyletic.

**Conclusions:**

Climatic oscillations over the Pleistocene probably played a major role in shaping the genetic diversity within this species complex. Different factors can explain their diversification, including Pleistocene forest refuges, riverine barriers and differentiation along environmental gradients. The earliest postulated members of the complex originated in central/eastern Africa and the first radiations took place in rain forests of the Congo Basin. A dramatic shift in the ecological requirements in early members of the complex, in association with changing environments, took place sometime after 1.13 Ma. Some lineages then colonized a substantial portion of the African continent, including a variety of savannah and forest habitats. The low genetic divergence of certain populations, some in isolated localities, can be explained by their synanthropic habits. This study underlines the need to revise the taxonomy of the *C. olivieri* complex.

**Electronic supplementary material:**

The online version of this article (doi:10.1186/s12862-015-0344-y) contains supplementary material, which is available to authorized users.

## Background

Climatic oscillations during the Pleistocene are known to have had a dramatic role in shaping the diversity and distribution of many African plant and animal species [1,2]. Several large-scale studies that tested this hypothesis have been carried out on large African mammals [3-5]. However, these animals are vagile and often broadly disperse or have large home ranges. Therefore, small mammals are excellent models for testing patterns of pan-African biogeography. There have been a few studies on sub-Saharan rodents [6-8], but shrews (Soricidae), a group known to have been used elsewhere to test Pleistocene climate oscillation models [9], have not been examined across broad ecological areas on the continent. These animals have short life spans, rapid reproduction cycles, low dispersal abilities and respond quickly to environmental changes [10], and are therefore a good model to investigate climate-driven models of diversification. All available genetic studies of African shrews focussed on taxa with relatively small geographical distributions (e.g. [9]). Members of the *Crocidura olivieri* (Lesson, 1827) complex [11], which have notably large body size for shrews and can weigh up to 65 g [12], are a rare example of a widespread Afrotropical soricid taxon. Members of the *C. olivieri* complex occur in a variety of habitats, including tropical rain forests, marshes, savannah and montane areas [12]. Therefore, this group is a potentially excellent model to test how climatic oscillations and associated changes in vegetation during the Pleistocene influenced their distribution and diversification.

Models employed to understand diversification events within vertebrates faunas are too numerous to review in detail here. By and large, hypotheses concerning factors that promote speciation in tropical faunas have been preoccupied with the geographical context of speciation and fall into two categories: allopatric and parapatric models. With allopatric models, extrinsic barriers to gene flow lead to the separation of sub-populations, which evolve differently associated with genetic drift and natural selection. For example, the “Pleistocene forest refuge” hypothesis, originally formulated for tropical South America and then applied to the Afrotropics, postulates that during the last 2.5 Myr, climatic oscillations caused phases of contraction and expansion of different vegetation types [1]. Severe and long-lasting dry and cold periods reduced forests to isolated remnants or ‘refuges’ allowing diversification and allopatric speciation [1]. The “riverine barrier” hypothesis argues that rivers acted as physical barriers associated with the distribution of certain taxa, promoting diversification [13]. Allopatric models have been invoked to explain the current distribution and diversity of African small mammal species [10,14-16]. The gradient model of diversification (parapatric or ecotonal model) has been proposed as an alternative to allopatric models [17]. It suggests that strong environmental gradients result in adaptive divergence and speciation for taxa tolerant of a broad range of habitats. A recent study has emphasized the role of habitat gradients in lineage diversification in the African rodent *Cricetomys* [7].

The taxonomy of the C. *olivieri* complex has been discussed for almost a century but uncertainties remain. Species currently considered valid within the C*. olivieri* complex are distributed across different habitats: C*. goliath* Thomas, 1906 is endemic to rain forests of the Congo Basin, *C. viaria* (I. Geoffroy, 1834) and *C. fulvastra* (Sundevall, 1843) are encountered in the Sudanian savannah from Morocco to Kenya and from Mali to Ethiopia, respectively, and C*. olivieri* is widespread across portions of the African continent [12].

**Figure 1:**
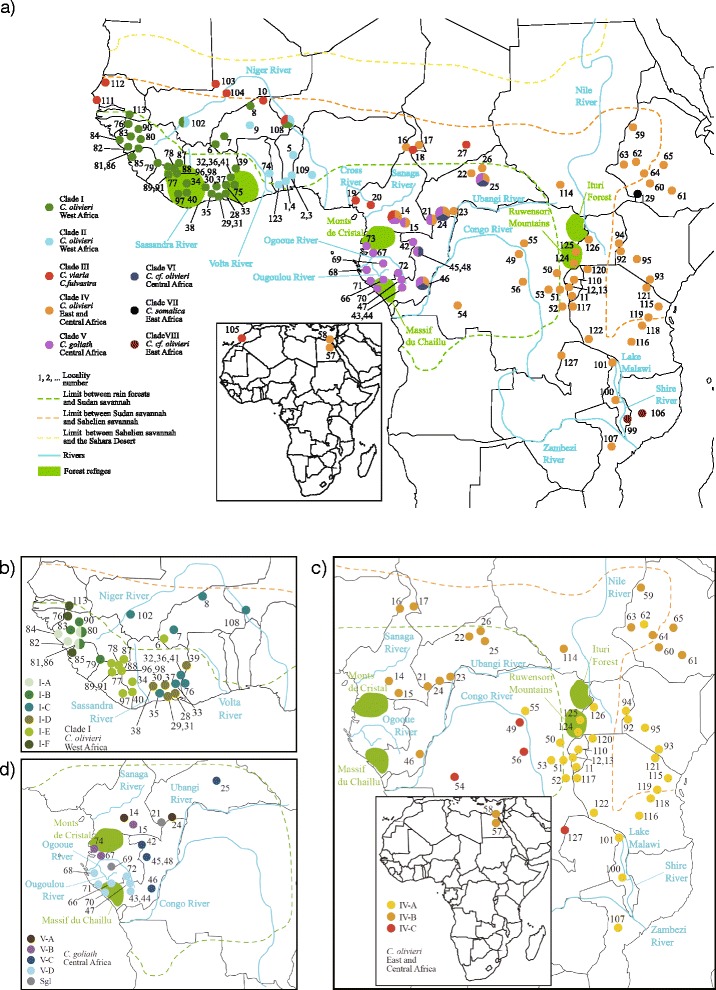
Sampling points and clade distributions. Map of sampling points showing the distribution of the phylogenetic clades identified in the Bayesian phylogenetic analyses: 8 main clades of the clade VII: *C. somalica and* clade VIII: *C. hera* complex **(a)**, subclades within clade I **(1b)**, subclades within clade IV **(c)** and subclades within clade V **(d)**. Ecological divisions based on White’s (1981) vegetation map. See Additional file 6 for localities names and GPS coordinates.

The specific aims of this study are: 1) to provide a greater understanding of the systematics of African giant shrews of the *C. olivieri* complex, specifically specimens identified based on morphology as *C. olivieri*, *C. viaria*, *C. fulvastra* or *C. goliath*; 2) to test for the respective roles of forest retraction/expansion, rivers (allopatric models), ecological gradients (parapatric model) and anthropogenic factors in explaining the diversification and current distribution of the different lineages. In order to infer biogeographical scenarios at the scale of the African continent and to answer questions concerning possible factors promoting diversification within the complex, we used data from three mitochondrial and four nuclear markers and conducted phylogenetic and population genetics analyses on animals originating from most of its geographical range (Figure 1).

## Results

### Phylogenetic relationships

**Figure 2:**
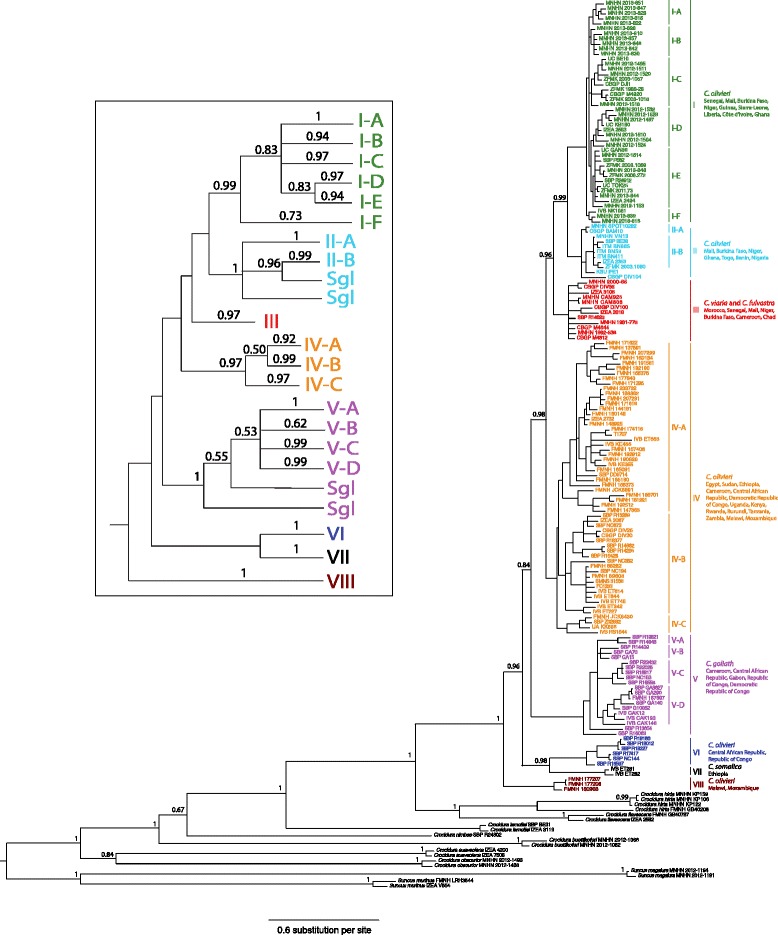
Phylogenetic tree of the *Crocidura olivieri* complex. Final phylogenetic tree for members of the *Crocidura olivieri* complex using one specimen per locality and per clade, with 156 individual specimens. Tree built using Bayesian Inference and data from three mitochondrial (16S, cytb and COI) and four nuclear markers (BRCA1, STAT5A, HDAC2 and RIOK3) for a combined total of 5305 bp. Values above branches are Bayesian posterior probabilities. The branchlets are identified by specimen numbers defined in Additional file 6. A simplified representation of the tree is shown in the upper framed window (Sgl = singleton).

Based on Bayesian Inference of combined data from the seven markers (Figure 2 and Additional file 1) the *C. olivieri* complex (specimens morphologically identified as *C. olivieri*, *C. viaria*, *C. fulvastra C. goliath* or *C. somalica*) forms a highly supported monophyletic group (pp = posterior probability > 0.95). This complex is the sister clade to a clade composed of *C. flavescens* and *C. hirta*. The clade composed of these seven species is reciprocally monophyletic with respect to *C. lamottei*. Within the *C. olivieri* complex, eight main clades can be identified. Clades I, II, IV, VI and VIII are composed of specimens assigned to *C. olivieri* based on morphology. Specimens assigned to *C. viaria* and *C. fulvastra* are gathered in clade III but do not form two monophyletic groups. Phylogenetic relationships are poorly resolved within this clade. Clade V comprises all specimens originally identified as *C. goliath* and clade VII the two specimens morphologically identified as *C. somalica*. Clades I + II, I-to-III, I-to-IV, VI + VII and I-to-VII are strongly monophyletic. Clade I-to-V is not supported (pp = 0.84).

The topology obtained through analysis of mtDNA data (Additional files 2 and 3) is very similar to the former, some nodes not recovered (monophyly of clade I-to-VII) or only poorly supported (clades VI + VII and I-to-III). Based on nuclear data (Additional files 4 and 5), the specimens referred to *C. olivieri*, *C. viaria*, *C. fulvastra*, *C. goliath*, *C. somalica*, *C. flavescens* and *C. hirta* form a monophyletic group. In the *C. olivieri* complex, only clades VI (pp = 0.90) and VIII (pp = 0.99) can be retrieved (specimens of clade VII could not be included because the nuclear markers did not be amplify). The other clades (I to V) were not recovered, presumably because of low variability in the nuclear data. *Crocidura hirta* forms a highly supported clade that is reciprocally monophyletic with *C. flavescens*.

No mtDNA haplotype was shared between clades I to VIII. Several main clades shared between them a single nuclear haplotype: clades I, II and III for BRCA1; clades I and III for RIOK3; clades I and II, clades IV and V, clades IV and VIII and clades I, II and IV for HDAC2; and clades I, II, III, IV and V, clades IV and VI and clades IV and V for STAT5A.

### Phylogeography of the *C. olivieri* complex

Individual specimens from the eight main clades in the C*. olivieri* complex can occur in sympatry, especially in central Africa (Figure 1). Six subclades can be identified within clade I, but phylogenetic relationships are not resolved. Clade I is composed of specimens obtained at forest or mixed forest-savannah sites (except subclade I-C also found in savannah) of western Africa (Senegal, Guinea, Liberia, Côte d’Ivoire, western Ghana, southern Niger, Burkina Faso and southwestern Mali), including individuals trapped in houses. Individuals placed in subclades I-C and I-D (pp = 0.97) occur on the left bank (following the watercourse from upstream to downstream) of the Sassandra River in western Côte d’Ivoire and specimens of subclade I-E (pp = 0.94) on the right bank (except one specimen captured further north). Clades I-D and I-E are sister clades with a low node support value (pp = 0.83).

Clade II includes specimens, which were trapped in the Sudanian savannah zone, north of the Guinean forest block and in the Dahomey Gap in Ghana, Benin, Togo, Nigeria, southern Niger, Burkina Faso and southwestern Mali. Clade III encompasses all sequenced individuals identified as C. *viaria* (Morocco, Senegal, southwestern Niger, northern Burkina Faso and southern Cameroon) and *C. fulvastra* (Chad and northern Cameroon); these specimens were collected on the northern fringe of the Sudanian and Sahelian savannah zones, except for animals from southern Cameroon obtained in forests, croplands and oil palm plantations.

Clade IV is composed of specimens obtained in a broad range of habitats (forest, cropland, garden and savannah). The geographic range of this species group extends throughout central and eastern Africa with isolated localities in Egypt (the type locality of *C. olivieri*). The subclade IV-A (pp = 0.92) is composed of specimens from the east and southeast of the continent, all of them from the right bank of the Congo River (Figure 1). The northern subclade IV-B (pp = 0.99) is encountered in Cameroon, CAR, eastern Congo, Egypt, Ethiopia and southern Sudan, on the right bank of the Congo and Ubangi Rivers. The subclade IV-C (pp = 0.97) is found in DRC and northeastern Zambia, on the left bank of the Congo River, except for specimens from the Ntumbachushi Falls (loc. 128). Clades IV-A and IV-B appear as sister clades but this relationship is not supported (pp = 0.50).

Specimens of clade V originate from the central African rain forests on the right bank of the Ubangi-Congo River. Subclades V-B (southern Cameroon and northern Gabon; pp = 0.62) and V-D (southern Gabon and southwestern Congo; pp = 0,99) + specimen R16063 (Makande, Gabon) exhibit an allopatric distribution on each side of the Ogooué River: subclade V-B occurs on the left bank and subclade V-D and the isolated specimen on the right bank. Makande is situated between the Ogooué River and one of its tributaries, the Ougoulou River. The individuals representing clade VI originate from Congo and CAR rain forest. Several subclades are observed within this clade but specimens do not cluster by their geographical positions. They are largely sympatric with *C. goliath* (clade V).

Specimens of clade VII originate from mesic places in the Mago National Park in southwestern Ethiopia, where they were trapped in plain savannah with dense shrubs. Clade VIII is composed of specimens from northern Mozambique and southern Malawi that were obtained in montane forest ‘islands’ including gallery forests. This clade is bounded by the Shire River to the west, the Zambezi River to the south, open savannah woodland to the east and Lake Malawi to the north.

### Genetic distances

The mean percentage of cytb sequence K2P divergence between various clades of the complex ranges from 1.34% (between clades I and II) to 4.90% (between clades V and VII). Within clades, the mean percent divergence ranges from 0.32% (clade VII) to 1.78% (clade IV) (Table 1a).

**Table 1:** **Genetic distances within the** ***Crocidura olivieri*** **complex**

	**I**	**II**	**III**	**IV**	**V**	**VI**	**VII**	**VIII**
**a)**								
I	**0.45** [0–1.0]	**1.34**	**1.50**	**2.26**	**4.13**	**3.98**	**4.43**	**3.51**
	**0.54** [0–1.6]	[0.6-2.1]	[0.9-3.0]	[1.2-3.4]	[2.9-5.2]	[3.2-4.7]	[3.5-5.6]	[3.0-4.2]
II	**1.61**	**0.51** [0–1.4]	**2.01**	**2.70**	**4.48**	**4.26**	**4.78**	**3.99**
	[1.0-2.7]	**0.56** [0–1.7]	[1.1-3.0]	[1.5-3.5]	[2.8-5.8]	[3.0-5.2]	[4.1-5.5]	[3.0-4.7]
III	**1.91**	**2.35**	**1.12** [0–1.9]	**2.43**	**4.36**	**4.35**	**4.82**	**3.79**
	[1.0-3.6]	[1.5-4.2]	**1.40** [0–2.9]	[1.6-3.4]	[3.1-5.2]	[3.5-5.3]	[3.6-6.8]	[3.1-4.6]
IV	**2.62**	**2.92**	**2.82**	**1.78** [0–2.7]	**4.65**	**4.58**	**4.78**	**3.98**
	[1.5-4.2]	[2.1-4.3]	[1.6-4.2]	**1.95** [0–4.0]	[3.3-5.4]	[3.5-5.4]	[4.1-5.6]	[3.2-4.5]
V	**4.52**	**4.69**	**4.75**	**4.67**	**1.26** [0.1-2.3]	**4.78**	**4.90**	**4.79**
	[3.5-5.7]	[3.9-6.6]	[3.5-6.0]	[3.5-6.0]	**1.12** [0–2.4]	[4.1-5.8]	[4.2-5.6]	[4.1-5.6]
VI	**4.54**	**4.66**	**4.91**	**4.83**	**4.92**	**0.65** [0.5-0.8]	**3.89**	**4.09**
	[3.5-5.6]	[4.0-5.7]	[4.0-6.4]	[3.9-6.6]	[4.0-6.2]	**0.63** [0–1.5]	[3.6-4.4]	[3.9-4.3]
VII	**4.22**	**4.57**	**4.60**	**4.57**	**4.68**	**3.75**	**0.32**	**3.58**
	[3.4-5.2]	[4.0-5.2]	[3.4-6.4]	[3.9-5.3]	[4.0-5.2]	[3.4-4.1]	**0.30**	[3.4-3.9]
VIII	**3.92**	**4.19**	**4.20**	**4.08**	**4.69**	**4.14**	**3.45**	**0.47**
	[2.3-5.1]	[3.8-5.0]	[3.3-5.9]	[3.3-5.3]	[3.9-5.4]	[3.8-5.1]	[3.2-3.8]	**0.47**
**b)**								
I	**0.75** [0–1.4]	**2.59**	**2.80**	**3.28**	**3.93**	**5.23**	**5.09**	**4.82**
	**0.75** [0–1.4]	[1.4-3.4]	[0.5-3.7]	[1.6-4.6]	[1.6-6]	[3.8-5.7]	[3.3-5.8]	[3.8-5.3]
II	**2.54**	**0.65** [0–1.6]	**2.15**	**2.80**	**4.62**	**4.48**	**4.30**	**4.65**
	[1.4-3.3]	**0.63** [0–1.5]	[1.2-3.0]	[1.6-4.1]	[3.5-5.2]	[4.0-4.8]	[3.6-4.8]	[4.0-5.2]
III	**2.79**	**2.10**	**0.49** [0–1.0]	**2.37**	**4.24**	**3.71**	**3.66**	**4.02**
	[1.4-3.6]	[1.2-2.9]	**0.49** [0–1.0]	[1.6-3.8]	[3.5-4.9]	[3.5-4.0]	[3.1-4.3]	[3.8-4.5]
IV	**3.23**	**2.77**	**2.37**	**1.39** [0–3.0]	**4.59**	**4.06**	**3.78**	**4.47**
	[1.5-5.8]	[1.5-4.9]	[1.7-4.9]	**1.31** [0–2.8]	[3.0-6.0]	[3.5-5.0]	[3.0-4.8]	[3.3-5.5]
V	**4.91**	**4.46**	**4.08**	**4.44**	**1.20** [0–2.2]	**4.55**	**4.45**	**4.43**
	[3.5-5.7]	[3.4-4.9]	[3.4-4.7]	[3.1-5.7]	**1.15** [0–1.7]	[4.0-5.1]	[3.8-5.0]	[3.7-5.1]
VI	**4.92**	**4.28**	**3.74**	**4.06**	**4.38**	**0.21** [0–0.3]	**2.16**	**4.10**
	[3.5-5.6]	[3.4-4.9]	[3.4-4.6]	[2.9-5.4]	[3.8-4.8]	**0.34** [0–1.1]	[2.0-2.2]	[4.1-4.1]
VII	**4.88**	**4.14**	**3.54**	**3.68**	**4.30**	**2.84**	**0.30**	**3.60**
	[3.2-5.5]	[3.5-4.6]	[3.0-4.1]	[2.9-4.6]	[3.8-4.8]	[2.0-4.6]	**0.30**	
VIII	**4.61**	**4.42**	**3.86**	**4.31**	**4.27**	**4.04**	**3.50**	**0.20**
	[3.7-5.1]	[3.8-4.9]	[3.7-4.3]	[3.2-5.2]	[3.5-4.9]	[3.8-4.4]		**0.20**

Genetic distances (K2P above and uncorrected p-distances below diagonal) between and within clades of the *C. olivieri* complex for cytb (1a) and COI markers (1b) with mean and range within brackets.

For COI, mean percentages between clades ranges from 2.15% (between clades II and III) to 5.23% (between clades I and VI). Within clades, the mean percent divergence ranges from 0.20% (clade VIII) to 1.39% (clade IV) (Table 1b).

### Divergence time estimates

**Figure 3:**
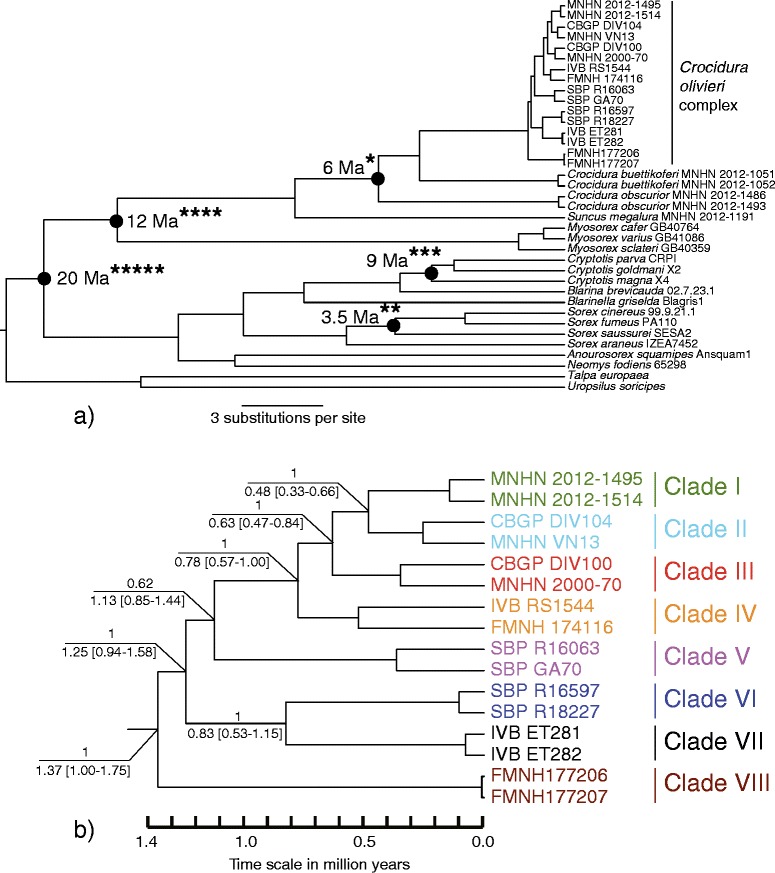
Datation tree of the *Crocidura olivieri* complex. Dating tree built using data of the three mitochondrial markers (16S, cytb and COI) based on 2403 bp and Bayesian Inference under a Yule model of speciation and a log-normal relaxed molecular clock model. **a)** Complete tree showing the four fossil calibration points used as time constraints: oldest record of *Crocidura* (*), oldest *Otisorex* (**), oldest *Cryptotis* (***), oldest recorded Myosoricinae-Crocidurinae (****) and split between Soricinae and Crocidurinae-Myosoricinae (*****). **b)** Focus on the *C. olivieri* complex with notations at nodes of Bayesian posterior probabilities (above line) and estimated times of divergence and credibility intervals within brackets (below line).

Phylogenetic relationships between clades I to VIII inferred from divergence time estimates analysis using BEAST were the same as those obtained with Bayesian analysis based on concatenated data from the seven markers. The time to the most recent common ancestor of the C*. olivieri* complex is estimated after 1.37 Ma (highest posterior density (HPD) interval containing 95% of the sampled values: 1.00-1.75, Figure 3). This corresponds to the split leading to the separation of clade VIII. The bifurcation between clades I-to-V and VI + VII is dated after 1.25 Ma (0.94-1.58) and that between clade V and I-to-IV after 1.13 Ma (0.85-1.44). The most recent divergence event corresponds to the split between clades I and II and is dated after 0.48 Ma (0.33-0.66).

Divergence time estimates inferred using a standard mammalian mutation rate of 2.2% per Myr [18] are between 16.7 and 31.7% lower than those obtained with fossil calibration points. The time to the most recent common ancestor of the complex is estimated after 1.09 Ma (0.90-1.29). The bifurcation between clades I-to-V and VI + VII is dated after 1.01 Ma (0.85-1.19) and that between clade V and I-to-IV after 0.92 Ma (0.75-1.08). The most recent divergence event corresponds to the split between clades I and II and is dated after 0.40 Ma (0.30-0.51). All these estimates fall within 95% intervals inferred from the analysis using calibration points. We therefore chose to base our biogeographic scenario hypotheses on fossil calibrations.

### Historical demography

Based on mitochondrial data (16S, cytb and COI), the nucleotide diversity (×100) ranges from 0.296 (clade VI) to 1.584 (clade IV) with a decrease from clade IV to clade I. Haplotype diversity ranges from 0.673 (clade VI) to 0.993 (clade V) with a decrease from clade V to clade II (Table 2). Based on mismatch distributions, a signal of demographic expansion was detected for all clades except for clade VI. The mode of the curve (tau) showed the highest value for clade IV, followed by clade V and the lowest value for clade I. A signal of spatial expansion was detected for all clades. The Fu’s *Fs* neutrality tests indicated significant population growth for clades I, IV and V. A spatial expansion was detected for clades IV (significant *Fs*, whereas F* and D* were not significant). Extended skyline plot analyses revealed demographic growth for clades I, III and IV based on numbers of population changes (1 and 2 for clade I, 1 for clade III and 1, 2 and 3 for clade IV) [19].

**Table 2:** **Genetic structure indices within the** ***Crocidura olivieri*** **complex**

**Clade**	**N**	**Np**	**Nh**	**Hd**	**Pi**	**k**	**Mismatch distribution goodness-of-fit test (P)**			**Fu and Li’s D test**	**Fu and Li’s F test**	**Fu’s Fs test**	**Tajima’s D test**
							**Dem exp**	**Tau**	**Spatial exp**				
I	90	115	59	0.969	0.447	9.426	**0.25**	**10.4**	**0.41**	−2.08	**−2.45**	**−24.57**	**−1.95**
										*P* > 0.05	***P*** **< 0.05**	**ss = 1.00**	***P*** **< 0.05**
II	17	37	11	0.882	0.473	9.868	**0.41**	**23.6**	**0.65**	0.37	0.17	0.62	−0.40
										*P* > 0.1	*P* > 0.1	ss = 0.06	*P* > 0.1
III	17	56	14	0.978	0.783	16.63	**0.35**	**20.2**	**0.75**	0.15	0.13	−1.41	0.017
										*P* > 0.1	*P* > 0.1	ss = 0.93	*P* > 0.1
IV	95	276	72	0.991	1.584	33.87	**0.85**	**41.6**	**0.99**	−1.06	−1.42	**−14.43**	−1.25
										*P* > 0.1	*P* > 0.1	**ss = 1.00**	*P* > 0.1
V	37	158	33	0.993	0.911	19.72	**0.23**	**24.4**	**0.48**	**−2.98**	**−3.06**	−**8.21**	**−1.79**
										***P*** **< 0.05**	***P*** **< 0.05**	**ss = 1.00**	***P*** **< 0.05**
VI	11	19	4	0.673	0.296	6.582	0.009	-	**0.44**	−0.40	−0.32	4.35	0.07
										*P* > 0.1	*P* > 0.1	ss = 0.06	*P* > 0.1

Genetic diversity, mismatch distributions and neutrality estimates for the main clades of members of the *C. olivieri* complex identified in the phylogenetic analyses based on three combined mitochondrial markers (16S, cytb and COI). Data include number of specimens (N), number of polymorphic sites (Np), number of distinct haplotypes (Nh), haplotype diversity (Hd), nucleotide diversity (Pi, expressed as percentages, i.e. 0.001 = 0.1%) and average number of pairwise nucleotide differences (k). Values indicating a signal of demographic or range expansion are in bold (P-value = *P* < 0.05; ss = Strobeck’s statistic > 0.95 for Fu’s Fs test). Tau is the mode of the curve of the mismatch distribution when a signal of demographic expansion is detected. It is proportional to the time since expansion. Clades VII and VIII are not included owing to a low number of specimens (n = 2 and n = 3, respectively).

## Discussion

### Alternative models to explain speciation and diversification events within the *Crocidura olivieri* complex

#### Forest refuge theory

All divergence events within the C*. olivieri* complex occurred after 1.37 (1.00-1.75) Ma, i.e. when the periodicity of glacial cycles switched from 41,000 to 100,000 years and induced prolonged periods of aridity on Africa [2]. Divergence between clades I-to-V, VI, VII and VIII occurred around 0.83-1.37 (0.54-1.75) Ma. These dates should be interpreted with caution given the wide divergence time intervals. On the African continent, the period around 1 Ma was characterized by notable aridity coinciding with a reduction of lowland forests and an expansion of savannah biomes [20]. This period coincides with divergence events for other African forest mammals [9,15,21]. Given that clades V, VI and VIII include forest-dwelling animals (lowland forests for clades V and VI and montane forests for clade VIII) and that these clades have allopatric or parapatric distributions, most of the early divergence events within the complex are congruent with the “Pleistocene forest refuge” hypothesis [1]: several populations of the complex may have been isolated for long periods in distinct forest refuges, favouring allopatric diversification.

Several central Africa refuges have been identified and provide insight into the current distribution of clades V, VI and VIII. Two refuges were described in the western part of the Congo Basin, the first near the Massif du Chaillu in southern Gabon and southern Congo and the second near Monts de Cristal in northern Gabon, Equatorial Guinea and southern Cameroon [20,22]. Within C. *goliath*, the distributions of clades V-B (not supported) and V-D are coincident with the Monts de Cristal and Massif du Chaillu refuges, respectively. Clade VIII is composed of members from two montane inselbergs, one in southern Malawi (Mt Mulanje) and the second in northern Mozambique (Mt Namuli), in the Malawi Rift. This rift forms a southern extension of the Albertine Rift. Their ancient age of divergence is consistent with this section of the Rift Valley being an early isolate from the main section of the Albertine Rift Valley to the north (and west). Two refuges occur on the Albertine Rift near the border of DRC and Uganda: the Ruwenzori Mountains and Ituri Forest [20,22].

Clade I is essentially encountered in Guinean forests. The phylogenetic relationships remain unresolved for the six clades I-A to I-F, which exhibit largely disjunct, but in some cases overlapping geographical ranges. This pattern is congruent with an ancestral population having experienced rapid habitat fragmentation, such as forest retraction during arid climatic phases, resulting in lineage sorting and then divergence between allopatric populations [15]. The genetic structure and distribution of clade I show similar patterns to that of the rodent *Praomys rostratus* [15], in which the observed distribution of two sister clades I-D and I-E fits the hypothesized areas of two Pleistocene forest refuges, one in southwestern Ghana and the other in Côte d’Ivoire, Liberia and southeastern Guinea [9,20,22]. However, this hypothesis has to be considered with caution owing to the weak support of the monophyly of clade I-D + I-E.

#### Gradient model of diversification

The split between clade V and clades I-to-IV is dated after 1.13 Ma (0.85-1.44), which corresponds to a new geological period of aridity and forest retraction [2]. Diminishing forested habitats and the progression of savannah might have facilitated a niche displacement at the forest-savannah ecotone. As populations belonging to clade V are typically forest-dwelling, whereas those of clades I-to-IV are frequently trapped in savannah and secondarily in forests, an ecological gradient model of diversification [17] may be invoked to understand these divergence events. As the ancestral population of clades I to V probably inhabited central African forests (according to the topology of the different phylogenetic trees), dispersal is postulated to have occurred from forest habitat to a mosaic of habitats composed of forest-savannah, and then to savannah. This resulted in parapatric differentiation along an ecological gradient via natural selection.

The most recent successive splits of clades IV, III, II and I complex occurred after 0.78 (0.57-1.00), 0.63 (0.47-0.84) and 0.48 Ma (0.33-0.66). Each of these diversification events includes taxa currently showing parapatric distributions, with cases of syntopy at some localities. The distributions of these clades can be superimposed on the major African floristic zones (Figure 1) [23]: clade IV is recorded mainly from central African forests and croplands surrounded by a mosaic of forest and Sudanian savannah and woodlands of eastern Africa; clade III from drier Sudanian and Sahelian savannah; clade II from western African Sudanian savannah; and clade I mainly from Guinean forests. These geographical distributions occur along a geographical and ecological gradient and sister clades occupy adjacent and distinct habitats with some overlap; patterns typical of predictions from the gradient model of diversification [17]. It is hypothesized that the associated cladogenesis events occurred close to the current contact zones between these clades. These events may be related to the southward progression of the Sahara and Sahelian savannah during glacial cycles, as shown for *Taterillus* gerbils over the past 1 Myr [24]. Although estimates of divergence times need to be interpreted with caution, we can assume that climatic oscillations during the Pleistocene probably played an important role in the diversification of the C*. olivieri* complex, as already proposed for several African rodents [15,24].

#### Riverine barriers

Rivers can promote allopatric diversification by separating a population into two vicariant subpopulations that form sister clades [13]. Such patterns can be observed within the C*. olivieri* complex and have been described in other African mammals: clades I and II and the genus *Praomys* along the Volta River [16]; clades IV-A + IV-B vs IV-C and other mammal taxa along the Congo-Ubangi River [7,14]. This hypothesis should be considered with caution owing to the weak support of the monophyly of clade IV-A + IV-B.

Rivers can also restrict gene flow between populations that have diverged through other means. When rivers are the proximal, but not the causal mechanism of diversification, distinct genetic clades can be observed on opposite banks but are not necessarily sister clades. In agreement with the river barrier hypothesis, we found clades V and VI to be geographically restricted to the right bank of the Congo-Ubangi River and clade IV-C restricted to the left bank. The specimen from Makandé forms a singleton group and its distribution appears restricted to the area between the Ogooué and Ougoulou Rivers. The Ogooué River is known to constrain the distribution of the primate *Mandrillus sphinx* [21]. Clade V is only found south of the Sanaga River, which is the distribution limit for numerous mammal species [5,21]. Within clade I, the Sassandra River seems to limit gene flow between clades situated on each side (clades I-C and I-D on the left side and clade I-E on the right side), as already observed in other small mammal [9,15]. It seems likely that large modern watercourses have remained stable over the past 1 Ma with regards to their trajectory, but with variable water levels. Only few data are available concerning rivers’ histories, which are often traced using phylogeographical patterns [25].

### From a forest origin to a wide ecological success throughout Africa

#### Origin of the C. olivieri complex in central/eastern Africa and first radiation in the Congo Basin

Animals occurring within clades V to VIII, the most basal, are endemic either to eastern or central Africa, but the topology of the phylogenetic tree does not provide a precise signal as to the origin of the C. *olivieri* complex. Individuals associated with clades V and VI were captured in lowland rain forests of the Congo Basin, so the most likely scenario is that early members of the complex colonized this watershed. A radiation then occurred within forest ecosystems, probably correlated with increase in body size.

Only a few specimens of clades VI (n = 15), VII (n = 2) and VIII (n = 3) were available and a possible explanation of their rarity could be competition. Indeed, distribution of clade VI seems to be limited to the easternmost fringe of the distribution of clade V, i.e. *C. goliath*. No other basal lineage than clade VIII was found in the Albertine Rift and it can be speculated that they were replaced on most of Albertine and southern Rift mountains during Pleistocene by individuals of clade IV-A, which represent the expanding population of animals with larger body size. Clade VIII thus represents a relict population. Another explanation to the low effectives of clades VI to VIII could be some form of habitat segregation due to strict ecological requirements. Indeed, specimens of clade VII were trapped only in mesic places in shrubby savannah and specimens of clade VIII only along streams in dense montane forest.

#### Exit from forests approximately 1.13 Ma and colonization of other habitats

Specimens of clade I-to-IV are encountered in many habitats (forest, savannah, field, gardens and houses) of the African continent and from Senegal to Ethiopia and from Chad to South Africa, whereas clades V, VI and VIII would be restricted to Congolese or montane forests. These patterns support an important relaxing of habitat requirements after 1.13 (0.85-1.44) Ma and more precisely, a partial exit out of forest, congruent with the gradient model of diversification. This event may have dramatically expanded the dispersal abilities within the complex and could explain its subsequent ecological success across much of the African continent. A marked signal of recent population and range expansion was detected for clade IV. *Crocidura olivieri* (sensu stricto) is one of the most abundant shrew species in different small mammal communities [26,27].

#### Colonization of northern and western Africa and secondary return to a forest habitat

A pattern of colonization from the Congo Basin toward northern Africa and then western Africa is consistent with the asymmetric topology of the mitochondrial and concatenated trees (paraphyletic position of eastern and central African lineages that exhibit relatively longer branch lengths; [28]) and estimates of genetic diversity. A progressive decline in population nucleotide and haplotype diversity from central (clades IV and V) to western Africa (clades I and II) can be observed. Population expansion occurred earlier in central Africa (clade IV) than in western Africa (clades I to III). The colonization of western Africa is postulated to have started after 0.78 Ma (0.57-1.00) (split between clades IV and I-to-III) and the ancestral population of clade I-to-III may have then expanded westward along the Sahelian savannah corridor (Figure 1). Interestingly, the recently diverged clade I is essentially encountered in Guinean forests, while its sister clade (clade II) is restricted to the surrounding Sudanian savannah, which we interpret as a return to the original forest habitat.

#### The role of human movements in the dispersion of the C. olivieri complex

Specimens of clade I-to-IV were trapped in towns and villages and in some cases in houses (clades I and II). Owing to this previously reported synanthropic behaviour for members of the complex [29,30], human activities may have played a role in the dispersal of these animals. Specimens were captured in three isolated localities (Massa in Morocco, Kom Oshim and Abu Rawash in Egypt) but geographic distances are not correlated with genetic distances, i.e. these specimens show little genetic separation with respect to other representatives of their clades.

Other parallel examples are known from different African small mammals. Records of the synanthropic rodent *Arvicanthis niloticus* in the Sudanese Nile may be related to the expansion of early humans from central Africa northwards along the Nile Valley 177,000–64,000 years ago [31]. The rodent *Mus jotterandi* is thought to have migrated from sub-Saharan Africa to Morocco during the Middle Pleistocene (0.8-0.1 Ma) [32]. Similar patterns can be invoked to explain occurrence of the *C. olivieri* complex in northern Africa. The Sanaga River is recognized as a barrier to dispersal for several mammal taxa [5,21]. However, animals associated with clade III are recorded from both sides of this river. This clade probably originated in dry savannah and its distribution may be linked to human movements from northern dry Cameroon to the south, even across Sanaga.

### Insight into the systematics of the *C. olivieri* complex

Recent molecular data have shown paraphyly within C. *olivieri* and close phylogenetic relationships with C. *viaria* and C. *fulvastra*; all three taxa are also genetically close to *C. goliath* [11,33]. Our study sheds new light on the taxonomy of the complex. Based on a combination of mitochondrial and nuclear data, C. *goliath* from the Congo Basin appears to be monophyletic (clade V). *Crocidura olivieri* is polyphyletic and divided into five geographical clades showing allopatric or parapatric distributions. Molecular data from specimens collected close to the type locality of the nominate form (Sakkara, Egypt) were obtained (FMNH 68262 and 89608 from Kom Oshim and Abu Rawash, respectively) and their assignment to clade IV-B suggests that clade IV represents C*. olivieri* sensu stricto. Specimens of *C. viaria* and *C. fulvastra* are included in the well-supported clade III with parapatric distributions vis-à-vis other clades. Owing to the weakness of the phylogenetic signal, reciprocal monophyly of the two species could not be assessed. Dubey *et al*. already mentioned the paraphyly of *C. olivieri* with respect to *C. viaria* and *C. fulvastra* [11].

However, owing to aspects of sample size, number of base pairs employed and distribution of collection sites, Dubey *et al*. [11] did not recover the clades VI, VII and VIII and several nodes in their phylogeny were not well-supported. Genetic distances between clades I to IV range from 1.34% to 2.70% based on cytb data. These values fall within the range of intraspecific variation described for the genus *Crocidura* (0.4-8.9% [34]; 0-3.4% [35]) and well below interspecific variation (14.3-20.6% [34]; 5.5-23.5% [35]). Based on the COI marker, genetic distances between clades I to IV range from 2.15% to 3.28%. The range of intraspecific variation described for the genus *Crocidura* is 0-2.9% and that of interspecific variation 4.4-24.7% [35]. Our results therefore question the taxonomic status of C*. viaria* and C*. fulvastra*. On the other hand, the clades VI, VII and VIII may represent separate species, with the level of genetic divergence comparable to C*. goliath. Crocidura somalica* (clade VII), described from Middle Webi Shebeli near Geledi, Ethiopia, is part of the C. *olivieri* complex. On the basis of morphology, animals falling within clade VIII match the description of *C. occidentalis hera* Dollman, 1915 named from Shire Highlands, Malawi. This locality and localities 101 and 107 (clade VIII) are in close proximity and are isolated from adjacent landscapes by the Shire River to the west, the Zambezi River to the south and dry lowland savannah to the east. These results indicate that the C. *oliveri* complex is in need of a taxonomic revision, best accomplished with combined data on genetics, morphology and ecology.

## Conclusions

Our study sheds new light on the evolutionary history and genetic diversity of the C*. oliveri* complex. Using sampling encompassing the known geographical range of the complex, a combination of mitochondrial and nuclear data and several analyses (phylogenetic inference, genetic structure analyses and divergence time estimates), we propose an evolutionary scenario for this complex. We postulate an origin in central/eastern Africa followed by a colonization and radiation in rain forests of the Congo Basin. An emigration from this biotope took place approximately 1.13 Ma. Members of the complex then colonized eastern and western Africa across a considerable range of savannah and forest habitats. Cladogenesis events and changes in habitat and ecology were probably influenced by climatic oscillations during the last 1 Myr. We tested several models providing insights into underlying mechanisms associated with the diversification of the complex and found that a variety of factors can explain their current phylogeographic structure and distribution: Pleistocene forest refuges, riverine barriers, differentiation along environmental gradients and probable human related introductions.

This study reveals the need of taxonomic revision of the *C. olivieri* complex. We confirm the validity of C. *goliath* from the Congo Basin but, however, C*. olivieri* appears polyphyletic and divided into five geographical clades showing allopatric or parapatric distributions. We did not recover monophyly of C. *viaria* and C. *fulvastra* owing to their close genetic relationships. Values of genetic distances between clades I to IV (composed of specimens morphologically identified as C*. olivieri*, C. *viaria* and C. *fulvastra*) fall well below interspecific variation described among shrews, suggesting their merger into a single and widespread species. Clades VI, VII (C*. somalica*) and VIII may represent separate species, pending on a revision of available taxon names.

## Methods

### Biological material

For this study, we gathered 565 specimens (434 *C. olivieri*, 35 *C. viaria*, 13 *C. fulvastra*, 81 *C. goliath* and two *C. somalica* that had been identified based on morphological characters) from 128 localities on the African continent (Figure 1, Additional file 6). Our research was approved by the “Comité Cuvier d’éthique en matière d’expérimentation animale” (referral number 68.009). Animals were handled under the guidelines of the American Society of Mammalogists [36]. All samples were collected in strict compliance with local legislations, especially with the agreement of the Ministère des Eaux et Forêts in Côte d’Ivoire; the Ministère de la Recherche Scientifique et de l’Innovation in Cameroon; the Direction de la Faune et de la Chasse in Gabon; the Ministère de l’Environnement, des Eaux et Forêts et du Développement Durable in Guinea; the Wildlife Conservation Authority in Ethiopia; the Forest Service in Kenya; the Wildlife Authority in Zambia; the Institut National pour l’Environnement et la Conservation de la Nature in Burundi; the Department of Game and Wildlife Authority in Uganda; the Institut Congolais pour la Conservation de la Nature and the Centre de Recherche des Sciences Naturelles in DRC; the Wildlife Conservation Society and Development Board in Rwanda; and the Gorongosa Restoration Project in Mozambique. Specimens were not concerned by restrictions regarding the Convention on Biological Diversity and the Convention on the Trade in Endangered Species of Wild Fauna and Flora.

### Modern and ancient DNA extraction and amplification

Fresh tissues were stored in 95% ethanol. DNA was extracted from liver, kidney or intercostal tissues using the NucleoSpin96 tissue kit (Machery-Nagel). We amplified three mitochondrial markers, two being protein-coding, cytochrome oxidase I (COI, 702 bp) and cytochrome *b* (cytb, 1166 bp), and one ribosomal, 16S (535 bp); and four nuclear markers, one exon, Breast Cancer 1 (BRCA1, 888 bp) and three introns, Signal Transducer and Activator of Transcription 5A (STAT5A, 598 bp), Histone Deacetylase 2 (HDAC2, 617 bp) and RIO Kinase 3 (RIOK3, 799 bp) (see Additional file 7) following the protocol in [9].

The three mitochondrial markers were chosen for their demonstrated high resolution for shrews and mammals at the specific and intraspecific levels [35,37]. The four nuclear markers had already been tested with shrews [9,38,39]. The use of several genetically independent loci improves the efficiency and robustness of taxa identification based on molecular data [40] and allows the detection of problems such as incomplete lineage sorting, hybridizing/introgression, retention of ancestral polymorphism, and paralogy resulting from transfer of mitochondrial DNA gene copies to the nucleus [41].

The extraction protocol we employed for ancient DNA has been extensively used for various sources of degraded DNA [42]. Herein samples consisted of dried tissue scraped from the cranium and mandible (four specimens of *C. olivieri* from Egypt collected in the 1950s close to Sakkara, the type locality) or last phalanx from one digit from mounted specimens (one *C. olivieri* and one *C. fulvastra* from Chad, three *C. viaria* from Senegal). We only succeeded in amplifying the two mitochondrial markers 16S and COI using specific internal primers. PCR conditions were initial denaturation of 3 min at 95°C, then 45 cycles of 95°C 10 s, 60°C 10 s, 72°C 20 s, and a final extension step at 72°C for 7 min. To avoid contaminations with modern DNA, all these steps were performed on separate days and in an isolated room exclusively devoted to manipulations of ancient DNA.

Purification of PCR products and cycle-sequencing reactions of both DNA strands were performed at the Génoscope (Evry, France). Sequences were edited and assembled using Sequencher 4.9 (Gene Codes Corporation, Ann Arbor, MI, USA) and deposited in GenBank (see Additional file 6).

### Phylogenetic analyses

To investigate the genetic diversity within the C*. olivieri* complex and to test for its monophyly, closely related species were included in the sampling [11] (see Additional file 6). Trees were rooted by specifying *Suncus megalura* and *S. murinus* as outgroups. For each marker, alignment was performed using ClustalW [43] (see Additional file 8). In order to assess phylogenetic relationships, among the 565 specimens, 491 were sequenced for the 16S marker, 436 for cytb, 311 for COI, 92 for BRCA1, 57 for STAT5A, 45 for HDAC2 and 49 for RIOK3; the lower number of sequenced samples for certain markers was to reduce costs of laboratory work and also due to difficulties in amplifying nuclear markers, but nuclear sequences were obtained for almost all mtDNA-defined lineages. We first tested for signs of positive selection affecting the coding nuclear marker BRCA using Datamonkey server and the SLAC method [44]. We also used the GARD method [45] to detect potential signs of recombination in each nuclear alignment. Neither positive selection nor recombination was detected in our dataset (significance level of 0.01). Phylogenetic relationships were inferred for each marker separately using Bayesian Inference (MrBayes version 3.1 [46]) following procedures described by Jacquet *et al*. [9]. No well-supported conflict was observed between topologies, so we built a restricted data set, corresponding to the seven markers combined (5305 bp) by retaining only one specimen per locality and per clade (156 specimens, see Additional file 6). A partition for each marker was defined. We also performed analyses using mtDNA and nuclear data separately. The existence of potential haplotype sharing between main clades revealed by phylogenetic analyses was investigated for each marker using Arlequin 3.11 [47].

K2P [48] genetic distances on the complete cytb and COI sequences were calculated among all samples (Mega 5 [49]) to compare our values to those provided in the literature. Uncorrected p-distances were also calculated.

### Divergence time estimates

A direct calibration of a molecular clock within the *C. olivieri* complex was not possible, because of the lack of a fossil record of this group in Africa [50]. Therefore, we used five external calibration points treated as minimum age constraints: split between Soricinae and Crocidurinae-Myosoricinae 20 Ma [51], oldest recorded Myosoricinae-Crocidurinae representative 12 Ma [52], oldest record of *Crocidura* (*C. kapsominensis*) 6 Ma [53], oldest *Otisorex* 3.5 Ma [54] and oldest *Cryptotis* 9 Ma [55]. We used sequences of the three mitochondrial markers and no nuclear marker because of the deep coalescence of the latter. Two specimens of the *C. olivieri* complex were selected within each clade I to VIII revealed by phylogenetic analyses. Each of these two specimens belonged to divergent subclades. Several specimens belonging to Soricidae species were also included to use calibration points described above. Trees were rooted by specimens of *Talpa europaea* and *Uropsilus soricipes*.

Times of divergence and credibility intervals were inferred using a Bayesian analysis implemented with BEAST 1.6.2 [56] under a Yule model. We used a log-normal relaxed molecular clock model [57], because phylogenetic rates obtained from the external ancient calibrations could be inapplicable for dating recent events (<1–2 Mya) due to rate decay [58]. All fossil calibration ages were treated as lognormal distributions [57], except the divergence between Soricinae and Crocidurinae-Myosoricinae that was used as a constraint [59] and thus treated as normal distribution. Posterior distributions of parameters were approximated using two independent MCMC analyses of 100,000,000 generations each. The first 10% of trees were discarded, and a 50% consensus tree was constructed from the remaining trees. We checked similarity of results from the two runs, and samples were then combined. Convergence of the chains was checked using the program TRACER 1.3 [60]. To check compatibility of calibration points, a cross-validation was used by repeating the analysis several times, leaving out in turn one of the calibrations and comparing node ages estimations. The maximum divergence in these estimations was observed between the topologies obtaining after leaving out the *Cryptotis* (9 Ma) and *Otisorex* (3.5 Ma) calibration points, respectively. Values of divergence for nodes within the *C. olivieri* complex were comprised between 6.6 and 9.5%. The analysis was also performed using a standard mammalian mutation rate (2.2% per Myr [18]).

### Estimates of genetic diversity, genetic structure and historical demography

Genetic diversity, genetic structure and historical demography were estimated for the major clades revealed by our phylogenetic analyses using combined data from the three mitochondrial markers (267 specimens for which all three markers 16S, cytb and COI were available). Nuclear markers were not employed because of their much lower variability. Number of sequences, polymorphic sites, haplotypes and nucleotide differences, and the haplotype and nucleotide diversities were estimated using Arlequin 3.11.

Several procedures were undertaken to test the hypothesis of recent population growth. Mismatch distributions [61], Tajima’s *D* [62], Fu and Li’s F* and D* [63] and Fu’s *Fs* tests [64] were calculated using Arlequin 3.11 and DnaSP 4.50.2 [65], and following the procedure described by Nicolas *et al*. [15]. An extended skyline plot analysis using BEAST 1.6.2 was generated to estimate an expansion based on multiple loci.

## Availability of supporting data

The sequence data supporting the results of this article are available in the GenBank repository [http://www.ncbi.nlm.nih.gov/genbank]. Accession numbers are provided in Additional file 6.

## Additional files

Additional file 1:
**Concatenated tree file.** File containing the topology of the tree built using Bayesian Inference and data from seven markers (16S, cytb, COI, BRCA1, STAT5A, HDAC2 and RIOK3) in .tre format. Additional file 2:
**Mitochondrial topology.** Phylogenetic tree built using Bayesian Inference and data from three mitochondrial markers (16S, cytb and COI) for 156 specimens of the *C. olivieri* complex. Values above branches are Bayesian posterior probabilities. The branchlets are identified by specimen numbers defined in Additional file 6. Additional file 3:
**Mitochondrial tree file.** File containing the topology of the tree built using Bayesian Inference and data from three mitochondrial markers (16S, cytb and COI) in .tre format. Additional file 4:
**Nuclear topology.** Phylogenetic tree built using Bayesian Inference and data from four nuclear markers (BRCA1, STAT5A, HDAC2 and RIOK3) for 37 specimens of the *C. olivieri* complex. Values above branches are Bayesian posterior probabilities. The branchlets are identified by specimen numbers defined in Additional file 6. Additional file 5:
**Nuclear tree file.** File containing the topology of the tree built using Bayesian Inference and data from four nuclear markers (BRCA1, STAT5A, HDAC2 and RIOK3) in .tre format. Additional file 6:
**Information on specimens used in this study.** Specimens used for molecular analyses with details of localities (locality number based on Figure 1 and coordinates in decimal degrees), information on mitochondrial clade, number of sequenced individuals for the locality and GenBank accession numbers. Owing to difficulties in amplifying the whole cytb marker, internal primers were designed, leading sometimes to the submission of several sequence portions. Additional file 7:
**Markers used in this study.** Markers used in this study with specific primers designed to amplify cytb and COI markers. Additional file 8:
**Sequence alignments.** Sequence alignment in fasta format for each marker (16S, cytb, COI, BRCA1, STAT5A, HDAC2 and RIOK3) used in this study.
