# Supplementary figures and images for: Phylogeography and evolutionary history of the Crocidura olivieri complex (Mammalia, Soricomorpha): from a forest origin to broad ecological expansion across Africa
Source: BMC Evol Biol. 2015 Apr 23;15:71. doi: 10.1186/s12862-015-0344-y (PMC4422046; doi:10.1186/s12862-015-0344-y)

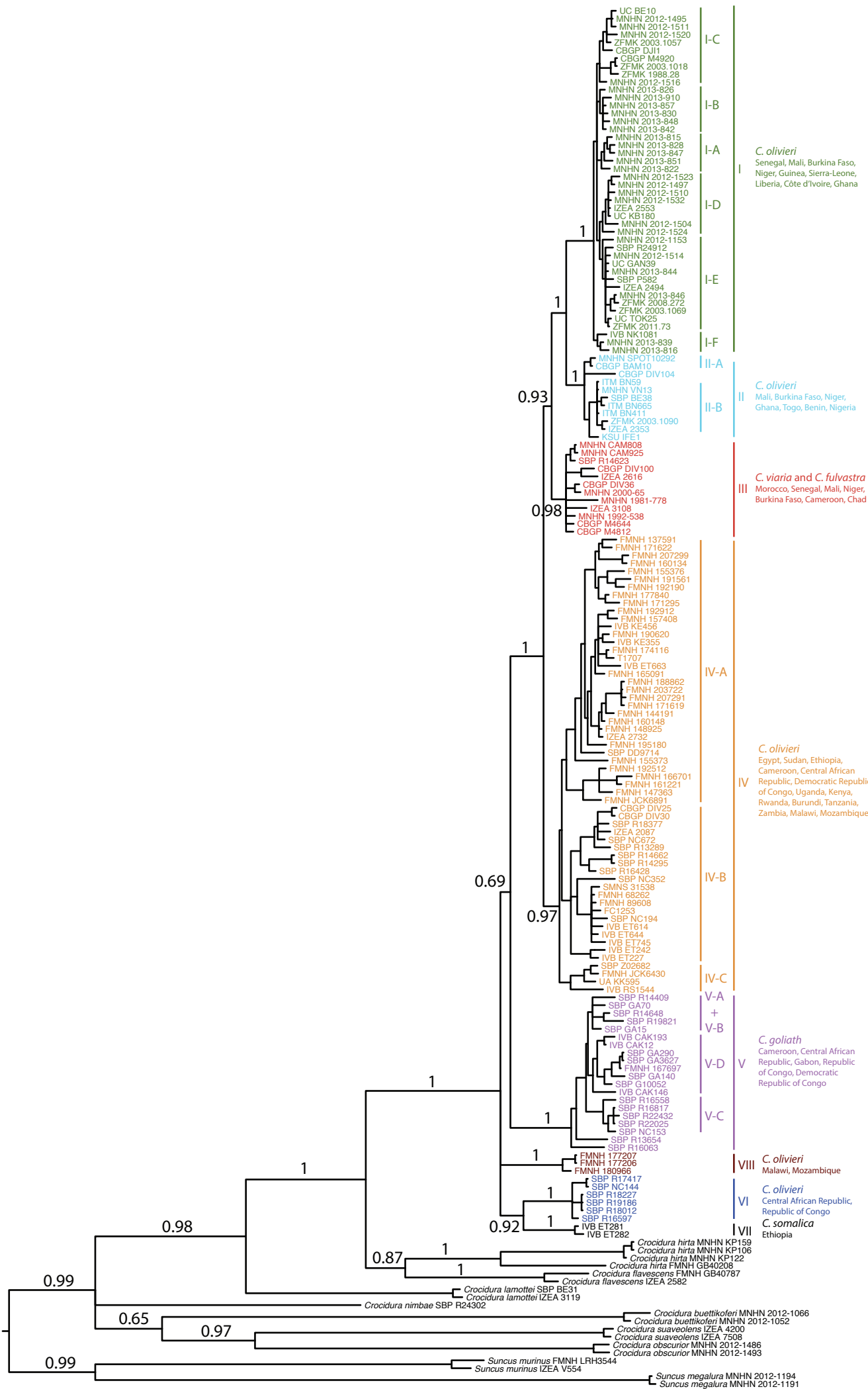

0.3 substitution per site

Supplement: Additional file 2: — Mitochondrial topology. Phylogenetic tree built using Bayesian Inference and data from three mitochondrial markers (16S, cytb and COI) for 156 specimens of the C. olivieri complex. Values above branches are Bayesian posterior probabilities. The branchlets are identified by specimen numbers defined in Additional file 6. [file 12862_2015_344_MOESM2_ESM.pdf]

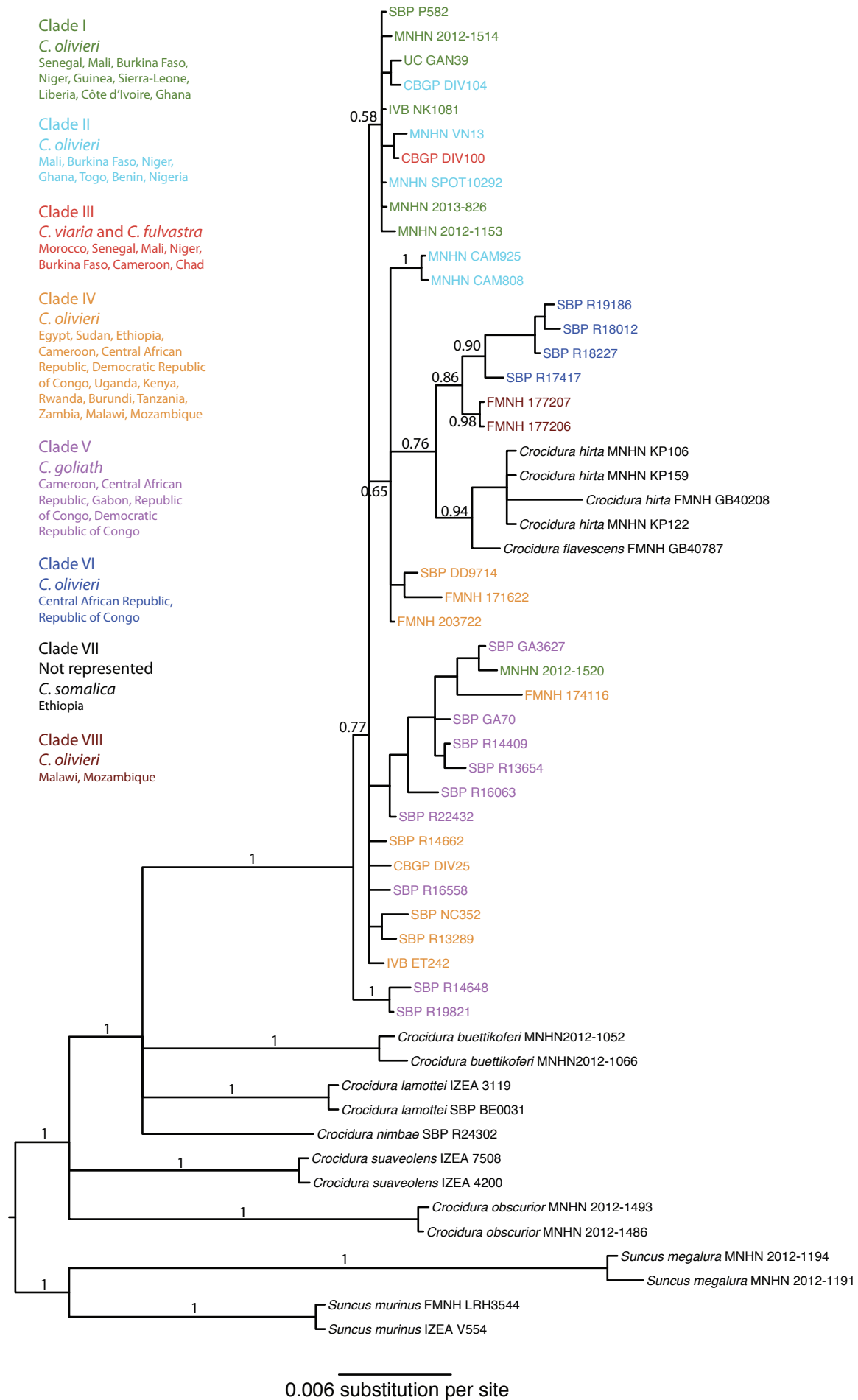

Supplement: Additional file 4: — Nuclear topology. Phylogenetic tree built using Bayesian Inference and data from four nuclear markers (BRCA1, STAT5A, HDAC2 and RIOK3) for 37 specimens of the C. olivieri complex. Values above branches are Bayesian posterior probabilities. The branchlets are identified by specimen numbers defined in Additional file 6. [file 12862_2015_344_MOESM4_ESM.pdf]
