# Supplementary material for: Phylogeography and evolutionary history of the Crocidura olivieri complex (Mammalia, Soricomorpha): from a forest origin to broad ecological expansion across Africa
Source: BMC Evol Biol. 2015 Apr 23;15:71. doi: 10.1186/s12862-015-0344-y (PMC4422046; doi:10.1186/s12862-015-0344-y)
Supplement: Additional file 6: — Information on specimens used in this study. Specimens used for molecular analyses with details of localities (locality number based on Figure 1 and coordinates in decimal degrees), information on mitochondrial clade, number of sequenced individuals for the locality and GenBank accession numbers. Owing to difficulties in amplifying the whole cytb marker, internal primers were designed, leading sometimes to the submission of several sequence portions. [file 12862_2015_344_MOESM6_ESM.doc]

**Additional file 6**

Abbreviations of collection codes: CBGP = Centre de Biologie pour la Gestion des Populations, Baillarguet, France, FMNH = Field Museum of Natural History, Chicago, USA, ITM = Institute of Tropical Medicine, Antwerp, Belgium, IVB = Institute of Vertebrate Biology, Studenec, Czech Republic, IZEA = Institut de Zoologie et d’Ecologie Animale, Lausanne, Switzerland, KSU = Kogi State University, Anyigba, Nigeria, MNHN = Muséum National d’Histoire Naturelle, Paris, France, SBP = Station Biologique de Paimpont, France, SMNS = Staatliches Museum für Naturkunde Stuttgart, Germany, UC = Université de Cocody, Adidjan, Côte d’Ivoire, UA = University of Antwerp, Belgium, ZFMK = Zoologisches Forschungsmuseum Alexander Koenig, Bonn, Germany.

Abbreviations of Countries: BE = Benin, BF = Burkina Faso, BU = Burundi, CAM = Cameroon, CAR = Central African Republic, CH = Chad, CHI = China, CI = Côte d’Ivoire, CON = Republic of Congo, DRC = Democratic Republic of Congo, EG = Egypt, ETH = Ethiopia, GAB = Gabon, GH = Ghana, GU = Guinea, IN = Indonesia, KE = Kenya, LB = Liberia, MA = Malawi, MAL = Mali, MO = Morocco, MOZ = Mozambique, NI = Niger, NIG = Nigeria, PHI = Philippines, RU = Russia, RW = Rwanda, SE = Senegal, SU = Sudan, TZ = Tanzania, TO = Togo, UG = Uganda, and ZA = Zambia.

| Taxon | Collection number | Country | Locality | Loc. number | Lat. (°) | Long. (°) | Clade | Number of spec. | 16S | Cytb | COI | BRCA1 | STAT5A | HDAC2 | RIOK3 |
| --- | --- | --- | --- | --- | --- | --- | --- | --- | --- | --- | --- | --- | --- | --- | --- |
| *C. fulvastra* | SBP R14623 | CAM | Bodjouo | 14 | 3.36 | 13.05 | III | 3 | AY675126 | KF690205 | KF684232 |  | KJ173694 | KJ173697 | KJ173699 |
| *C. fulvastra* | CBGP DIV36 | CAM | Meskine | 18 | 10.50 | 14.25 | III | 2 | KF684162 |  | KF684244 |  |  |  |  |
| *C. fulvastra* | MNHN CAM925 | CAM | Mundemba | 19 | 4.95 | 8.87 | III | 1 | KF684134 | KF690203 | KF684281 | KF757104 | KF757134 | KF757170 | KF757218 |
| *C. fulvastra* | MNHN CAM808 | CAM | Yabassi Ndgomen | 20 | 4.51 | 10.36 | III | 7 | KF684079 | KF690162 | KF684262 | KF757129 | KF757155 | KF757193 |  |
| *C. fulvastra* | MNHN 2000-65 | CH | Zakouma | 27 | 10.80 | 19.80 | III | 11 | EF524869 | EF524761 EF524655 DQ305261 |  |  |  |  |  |
| *C. goliath* | SBP R14648 | CAM | Bodjouo | 14 | 3.36 | 13.05 | V-A | 1 | JX312012 | KF690202 | JX312058 | KF757112 | KF757143 | KF757179 | KF757203 |
| *C. goliath* | SBP R14409 | CAM | Djomedjo | 15 | 3.08 | 13.60 | V-B | 1 | AY675154 | KF690178 | JX312057 | KF757124 | KF757152 | KF757192 | KF757221 |
| *C. goliath* | SBP R13654 | CAR | Adibori | 21 | 3.17 | 16.05 | V-Sgl | 1 | AF550861 | KF690186 | KF684210 | KF757127 | KF757167 |  | KF757223 |
| *C. goliath* | SBP R19821 | CAR | Batouri | 24 | 3.92 | 17.06 | V-A | 1 | AY675162 | KF690225 |  | KF757121 | KF757149 | KF757189 | KF757202 |
| *C. goliath* | SBP NC153 | CAR | Bohou | 25 | 7.72 | 21.33 | V-C | 1 | KF684180 |  |  |  |  |  |  |
| *C. goliath* | SBP R16817 | CON | Baie de l'ombrette | 42 | 1.07 | 14.48 | V-C | 2 | AF550849 | KF690154 | KF684225 |  |  |  |  |
| *C. goliath* | IVB CAK146 | CON | Bambama | 43 | -2.54 | 13.52 | V-D | 2 | KF684096 | KF690175 | KF684202 |  |  |  |  |
| *C. goliath* | IVB CAK12 | CON | Kissiki | 44 | -2.79 | 13.51 | V-D | 1 | KF684174 | KF690233 |  |  |  |  |  |
| *C. goliath* | SBP R22432 | CON | Mbomo | 45 | 0.58 | 14.85 | V-C | 33 | KF684147 | KF690157 | KF684236 | KF757113 | KF757163 | KF757180 | KF757201 |
| *C. goliath* | SBP R16558 | CON | Mbouambe | 46 | -2.90 | 15.65 | V-C | 7 | JX312014 | KF690173 | JX312060 | KF757128 | KF757168 |  | KF757225 |
| *C. goliath* | IVB CAK193 | CON | Simombondo | 47 | -2.32 | 13.66 | V-D | 3 | KF684087 | KF690167 | KF684199 |  |  |  |  |
| *C. goliath* | SBP R22025 | CON | Tchombi | 48 | 0.75 | 14.87 | V-C | 2 | KF684153 | KF690159 | KF684240 |  |  |  |  |
| *C. goliath* | FMNH 167697 | GAB | Doussala | 66 | -2.40 | 11.40 | V-D | 1 | KF684118 | KF690194 | KF684268 |  |  |  |  |
| *C. goliath* | SBP GA70 | GAB | Four-Place | 67 | -0.25 | 10.33 | V-B | 1 | KF684116 | KF690192 | KF684215 | KF757117 | KF757146 | KF757184 | KF757208 |
| *C. goliath* | SBP GA290 | GAB | Kili | 68 | -1.58 | 10.17 | V-D | 3 | KF684190 | KF699159 KF699193 | KF684205 |  |  |  |  |
| *C. goliath* | SBP R16063 | GAB | Makande | 69 | -0.68 | 11.92 | V-Sgl | 8 | AF550852 | KF690242 | KF684247 | KF757110 | KF757141 |  | KF757211 |
| *C. goliath* | SBP GA140 | GAB | Malounga | 70 | -3.15 | 10.75 | V-D | 1 | AY675161 | KF690236 |  |  |  |  |  |
| *C. goliath* | SBP GA3627 | GAB | Mbani | 71 | -2.41 | 10.50 | V-D | 7 | EF524866 | DQ305232 EF524758 EF524653 | KF684230 | EF525124 | KF757136 | KF757172 | KF757205 |
| *C. goliath* | SBP G10052 | GAB | Mbaya | 72 | -1.67 | 13.52 | V-D | 3 | AY675129 |  |  |  |  |  |  |
| *C. goliath* | SBP GA15 | GAB | Mvoum | 73 | 0.58 | 9.88 | V-B | 2 | AY675133 | KF690235 |  |  |  |  |  |
| *C. olivieri* | ITM BN59 | BE | Adjassagon | 1 | 7.00 | 1.95 | II-B | 4 | KF684113 | KF690184 | KF684263 |  |  |  |  |
| *C. olivieri* | ITM BN665 | BE | Ahozonnoude | 2 | 6.81 | 2.38 | II-B | 4 | KF684156 | KF690219 | KF684294 |  |  |  |  |
| *C. olivieri* | ITM BN411 | BE | Houedja | 3 | 7.14 | 2.44 | II-B | 2 | KF684173 | KF690232 |  |  |  |  |  |
| *C. olivieri* | MNHN VN13 | BE | La Lama | 4 | 7.00 | 2.10 | II-B | 10 | KF684151 | KF690215 | KF684237 | KF757116 | KF757164 | KF757183 | KF757215 |
| *C. olivieri* | SBP BE38 | BE | Parakou | 5 | 9.38 | 2.67 | II-B | 2 | AY675142 | KF690216 | KF684239 |  |  |  |  |
| *C. olivieri* | IZEA 2494 | BF | Banfora | 6 | 10.63 | -4.75 | I-E | 4 |  | DQ305236 |  |  |  |  |  |
| *C. olivieri* | ZFMK 2003.1018 | BF | Bobo Dioulasso | 7 | 11.17 | -4.28 | I-C | 7 | KF684169 | KF690230 |  |  |  |  |  |
| *C. olivieri* | CBGP DJI1 | BF | Djibo | 8 | 14.08 | -1.62 | I-C | 2 | KF684164 | KF690226 |  |  |  |  |  |
| *C. olivieri* | MNHN SPOT10292 | BF | Ouagadougou | 9 | 12.33 | -1.58 | II-A | 8 | KF684123 | KF690197 | KF684220 | KF757122 | KF757150 | KF757190 | KF757200 |
| *C. olivieri* | IZEA 2732 | BU | Kiguena | 11 | -4.17 | 29.50 | IV-A | 4 | EF524851 | DQ305239 EF524638 EF524745 |  | EF525109 |  |  |  |
| *C. olivieri* | FMNH 148925 | BU | Kirumura | 12 | -2.90 | 29.25 | IV-A | 2 | KF684101 | KF690180 | KF684257 |  |  |  |  |
| *C. olivieri* | FMNH 137591 | BU | Teza | 13 | -3.20 | 29.50 | IV-A | 2 | KF684078 | KF690161 | KF684249 |  |  |  |  |
| *C. olivieri* | SBP R14662 | CAM | Bodjouo | 14 | 3.36 | 13.05 | IV-B | 15 | AF550853 | KF690153 | KF684224 | KF757106 | KF757161 | KF757173 | KF757206 |
| *C. olivieri* | SBP R14295 | CAM | Djomedjo | 15 | 3.08 | 13.60 | IV-B | 1 | AY675124 | KF690158 | KF684248 |  |  |  |  |
| *C. olivieri* | CBGP DIV25 | CAM | Gamnaga | 16 | 10.50 | 13.70 | IV-B | 6 | KF684080 | KF699161 KF699195 | KF684195 | KF757123 | KF757151 | KF757191 | KF757220 |
| *C. olivieri* | CBGP DIV30 | CAM | Maga | 17 | 10.75 | 15.00 | IV-B | 1 | KF684178 | KF699176 KF699209 | KF684245 |  |  |  |  |
| *C. olivieri* | SBP R13289 | CAR | Adibori | 21 | 3.17 | 16.05 | IV-B | 9 | KF684100 | KF690179 | KF684204 | KC684195 | KF757159 | KF757194 |  |
| *C. olivieri* | SBP NC672 | CAR | Bangora | 22 | 8.08 | 20.35 | IV-B | 1 | AY675123 |  |  |  |  |  |  |
| *C. olivieri* | IZEA 2087 | CAR | Bangui | 23 | 4.33 | 18.50 | IV-B | 5 | EF524830 | EF524726 EF524617 |  | EF525088 |  |  |  |
| *C. olivieri* | SBP R18012 | CAR | Batouri | 24 | 3.92 | 17.06 | VI | 10 | KF684160 | KF690222 | KF684243 | KF757107 | KF757138 | KF757175 | KF757197 |
| *C. olivieri* | SBP R18377 |  |  |  |  |  | IV-B | 19 | KF684159 | KF690221 | KF684242 |  |  |  |  |
| *C. olivieri* | SBP R18227 |  |  |  |  |  | VI | 10 | KF684103 | KF690182 | KF684207 | KF757125 | KF757153 |  | KF757198 |
| *C. olivieri* | SBP R19186 |  |  |  |  |  |  |  | KF684126 | KF699184 KF699217 | KF684222 | KF757105 | KF757135 | KF757171 | KF757204 |
| *C. olivieri* | SBP NC144 | CAR | Bohou | 25 | 7.72 | 21.33 | VI | 1 | AY675121 | KF690240 |  |  |  |  |  |
| *C. olivieri* | SBP NC352 |  |  |  |  |  | IV-B | 8 | KF684089 | KF690152 | KF684200 | KF757130 | KF757156 |  |  |
| *C. olivieri* | SBP NC194 | CAR | Koumbala | 26 | 8.32 | 21.28 | IV-B | 1 | AY675122 |  |  |  |  |  |  |
| *C. olivieri* | IZEA 2553 | CI | Abidjan | 28 | 5.33 | -4.00 | I-D | 12 | EF524829 | EF524724 EF524615 DQ305246 |  | EF525086 |  |  |  |
| *C. olivieri* | UC KB180 | CI | Aboisso | 29 | 5.45 | -4.23 | I-D | 1 | KF684172 | KF699178 KF699211 |  |  |  |  |  |
| *C. olivieri* | MNHN 2012-1497 | CI | Ahouakro | 30 | 5.90 | -4.85 | I-D | 6 | KF684099 | KF699158 KF699192 | KF684203 |  |  |  |  |
| *C. olivieri* | MNHN 2012-1504 | CI | Azagny | 31 | 5.23 | -4.79 | I-D | 8 | KF684127 | KF699172 KF699189 | KF684223 | KJ173690 |  |  |  |
| *C. olivieri* | MNHN 2012-1510 | CI | Bakro | 32 | 6.85 | -3.30 | I-D | 1 | KF684110 | KF699162 KF699196 | KF684212 |  |  |  |  |
| *C. olivieri* | MNHN 2012-1511 |  |  |  |  |  | I-C | 4 | KF684170 | KF699179 KF699212 |  |  |  |  |  |
| *C. olivieri* | UC BE10 | CI | Besso | 33 | 6.06 | -3.71 | I-C | 4 | KF684077 | KF699157 KF699187 | KF684194 | KJ173688 |  |  |  |
| *C. olivieri* | MNHN 2012-1514 | CI | Blodi | 34 | 6.75 | -7.30 | I-E | 2 | JX312016 | JX311974 KF699207 | JX312051 | JX312022 | KF757137 | KF757174 | KF757207 |
| *C. olivieri* | MNHN 2012-1516 | CI | Gbagroube | 35 | 5.85 | -5.30 | I-C | 1 | KF684166 | KF699181 KF699214 |  |  |  |  |  |
| *C. olivieri* | MNHN 2012-1520 | CI | Kotdkossou | 36 | 7.15 | -3.20 | I-C | 6 | KF684141 | KF699175 KF699191 | KF684234 | KF757101 | KF757160 |  |  |
| *C. olivieri* | MNHN 2012-1523 | CI | Lamto | 37 | 6.13 | -4.89 | I-D | 3 | KF684131 | KF699168 KF699202 | KF684226 | KJ173693 |  |  |  |
| *C. olivieri* | MNHN 2012-1524 | CI | Menekre | 38 | 6.15 | -6.00 | I-D | 4 | KF684106 | KF690185 | KF684209 |  |  |  |  |
| *C. olivieri* | MNHN 2012-1532 | CI | Sanguiebo | 39 | 8.15 | -2.60 | I-D | 5 | KF684120 | KF690196 | KF684217 | KJ173691 | KJ173695 |  | KJ173698 |
| *C. olivieri* | SBP R24912 | CI | Tai National Park | 40 | 5.85 | -7.38 | I-E | 5 | AF550854 | KF699155 KF699185 | KF684193 |  |  |  |  |
| *C. olivieri* | MNHN 2012-1495 | CI | Touzouko | 41 | 6.75 | -3.50 | I-C | 2 | KF684121 | KF699166 KF699200 | KF684218 | KJ173689 |  |  |  |
| *C. olivieri* | SBP R17417 | CON | Mbomo | 45 | 0.58 | 14.85 | VI | 3 | KF684140 | KF690156 | KF684233 | KF757132 | KF757165 | KF757195 | KF757224 |
| *C. olivieri* | SBP R16597 | CON | Mbouambe | 46 | -2.90 | 15.65 | VI | 1 | KF684081 | KF690163 | KF684196 | KJ173692 | KJ173696 |  |  |
| *C. olivieri* | SBP R16428 |  |  |  |  |  | IV-B | 1 | KF684185 | KF699188 |  |  |  |  |  |
| *C. olivieri* | SBP Z02682 | DRC | Babusoko-Amadiabe | 49 | -0.07 | 25.28 | IV-C | 2 | KF684192 | KF690228 |  |  |  |  |  |
| *C. olivieri* | FMNH 171619 | DRC | Idjwi Island | 50 | -2.24 | 28.80 | IV-A | 4 | KF684148 | KF690212 | KF684283 |  |  |  |  |
| *C. olivieri* | FMNH 171622 |  |  |  |  |  |  |  | KF684143 | KF690208 | KF684276 | KF757126 | KF757154 |  | KF757222 |
| *C. olivieri* | FMNH 203722 | DRC | Itombwe Forest | 51 | -3.34 | 28.75 | IV-A | 1 | KF684094 | KF690174 | KF684259 | KF757118 | KF757166 | KF757186 | KF757216 |
| *C. olivieri* | FMNH 195180 | DRC | Kabobo forest | 52 | -5.00 | 29.00 | IV-A | 1 | KF684098 | KF690177 | KF684253 |  |  |  |  |
| *C. olivieri* | FMNH 188862 | DRC | Kalibina | 53 | -3.40 | 27.75 | IV-A | 1 | KF684093 | KF690172 | KF684256 |  |  |  |  |
| *C. olivieri* | UA KK595 | DRC | Kikwit | 54 | -5.00 | 18.83 | IV-C | 2 | KF684176 | KF690237 |  |  |  |  |  |
| *C. olivieri* | SBP DD9714 | DRC | Kisangani | 55 | 0.60 | 25.22 | IV-A | 8 | KF684135 | KF699170 KF699204 | KF684229 | KF757109 | KF757140 | KF757177 | KF757210 |
| *C. olivieri* | FMNH JCK6430 | DRC | Maniema | 56 | -2.64 | 25.34 | IV-C | 1 | KF684183 |  |  |  |  |  |  |
| *C. olivieri* | FMNH 89608 | EG | Abu Rawash | 57 | 30.08 | 31.10 | IV-B | 3 | KF684186 |  | KF684287 |  |  |  |  |
| *C. olivieri* | FMNH 68262 | EG | Kom Oshim | 58 | 29.33 | 30.90 | IV-B | 1 | KF684189 |  | KF684286 |  |  |  |  |
| *C. olivieri* | IVB ET614 | ETH | Alatish | 59 | 12.27 | 35.73 | IV-B | 2 |  | KM658990 |  |  |  |  |  |
| *C. olivieri* | IVB ET242 | ETH | Arba Minch | 60 | 6.03 | 37.57 | IV-B | 4 |  | KM658985 | KM658992 |  | KM658996 |  |  |
| *C. olivieri* | IVB ET227 | ETH | Bale | 61 | 6.85 | 39.89 | IV-B | 1 |  | KM658984 |  |  |  |  |  |
| *C. olivieri* | IVB ET644 | ETH | Didessa Bridge | 62 | 8.69 | 36.41 | IV-B | 1 |  | KM658988 |  |  |  |  |  |
| *C. olivieri* | IVB ET663 | ETH | Gambella Town | 63 | 8.24 | 34.59 | IV-A | 1 | KM658982 | KM658989 | KM658995 |  |  |  |  |
| *C. olivieri* | IVB ET745 | ETH | Jimma | 64 | 7.70 | 37.07 | IV-B | 1 |  | KM658991 |  |  |  |  |  |
| *C. olivieri* | f.c. 1253 | ETH | Zebich | 65 | 9.00 | 39.00 | IV-B | 1 |  | EU742597 |  |  |  |  |  |
| *C. olivieri* | ZFMK 2003.1090 | GH | Apesokubi | 74 | 7.60 | 0.38 | II-B | 2 | KF684177 | KF690238 |  |  |  |  |  |
| *C. olivieri* | ZFMK 2003.1057 | GH | Krokosua Forest | 75 | 6.61 | -2.85 | I-C | 1 | KF684165 | KF690227 |  |  |  |  |  |
| *C. olivieri* | MNHN 2013-839 | GU | Altou Fonkola | 76 | 12.36 | -13.46 | I-F | 7 | KF684095 | KF699160 KF699194 | KF684201 |  |  |  |  |
| *C. olivieri* | MNHN 2013-844 | GU | Bamakama | 77 | 7.71 | -9.26 | I-E | 1 | KF684122 | KF690155 | KF684219 |  |  |  |  |
| *C. olivieri* | MNHN 2013-846 | GU | Bhoita | 78 | 8.08 | -8.91 | I-E | 2 | KF684082 | KF690151 | KF684197 |  |  |  |  |
| *C. olivieri* | MNHN 2013-826 | GU | Denguedou | 79 | 8.49 | -10.44 | I-B | 1 | KF684083 | KF699156 KF699186 | KF684198 | KF757102 | KF757158 |  | KF757227 |
| *C. olivieri* | MNHN 2013-847 | GU | Gagal | 80 | 11.08 | -12.29 | I-A | 2 | KF684152 | KF690160 | KF684238 |  |  |  |  |
| *C. olivieri* | MNHN 2013-848 |  |  |  |  |  | I-B | 2 | KF684142 | KF699183 KF699216 | KF684235 |  |  |  |  |
| *C. olivieri* | MNHN 2013-828 | GU | Ganya | 81 | 10.06 | -12.55 | I-A | 3 | KF684133 | KF699171 KF699205 | KF684228 |  |  |  |  |
| *C. olivieri* | MNHN 2013-830 |  |  |  |  |  | I-B | 3 | KF684124 | KF699167 KF699201 | KF684221 |  |  |  |  |
| *C. olivieri* | MNHN 2013-851 | GU | Gayebombo | 82 | 10.13 | -13.59 | I-A | 1 | KF684137 | KF699180 KF699213 | KF684231 |  |  |  |  |
| *C. olivieri* | MNHN 2013-857 | GU | Koba | 83 | 11.16 | -13.29 | I-B | 8 | KF684108 | KF699173 KF699206 | KF684211 |  |  |  |  |
| *C. olivieri* | MNHN 2013-815 | GU | Mankountan | 84 | 10.53 | -14.45 | I-A | 6 | KF684112 | KF699163 KF699197 | KF684213 |  |  |  |  |
| *C. olivieri* | MNHN 2013-816 | GU | Mola | 85 | 9.31 | -12.91 | I-F | 4 | KF684157 | KF699182 KF699215 | KF684241 |  |  |  |  |
| *C. olivieri* | MNHN 2013-822 | GU | Molota | 86 | 9.93 | -12.83 | I-A | 3 | KF684114 | KF699164 KF699198 | KF684214 |  |  |  |  |
| *C. olivieri* | MNHN 2013-910 |  |  |  |  |  | I-B | 3 | KF684105 | KF699174 KF699208 | KF684208 |  |  |  |  |
| *C. olivieri* | ZFMK 2003.1069 | GU | Mount Béro | 87 | 8.14 | -8.57 | I-E | 3 | KF684084 | KF690164 | KF684250 |  |  |  |  |
| *C. olivieri* | MNHN 2012-1153 | GU | Mount Nimba | 88 | 7.64 | -8.34 | I-E | 14 | JQ732582 | JQ732356 KF699190 | JQ732236 | JQ732467 |  |  | KF757228 |
| *C. olivieri* | ZFMK 2008.272 | GU | Pic de Foko | 89 | 8.50 | -8.91 | I-E | 4 | KF684175 | KF690234 |  |  |  |  |  |
| *C. olivieri* | MNHN 2013-842 | GU | Satina | 90 | 11.43 | -12.36 | I-B | 2 | KF684184 | KF699177 KF699210 | KF684246 |  |  |  |  |
| *C. olivieri* | SBP P582 | GU | Ziama Forest | 91 | 8.35 | -9.22 | I-E | 25 | KF684161 | KF690223 | KF684290 | KF757103 | KF757133 | KF757169 | KF757196 |
| *C. olivieri* | IVB KE355 | KE | Kakamega | 92 | 0.24 | 34.86 | IV-A | 1 | KF684129 | KF690200 | KF684274 |  |  |  |  |
| *C. olivieri* | T1707 | KE | Kilimandjaro | 93 | -3.00 | 37.50 | IV-A | 2 | AY675130 |  |  |  |  |  |  |
| *C. olivieri* | IVB KE456 | KE | Kitale | 94 | 1.03 | 34.83 | IV-A | 2 | KF684102 | KF690181 | KF684206 |  |  |  |  |
| *C. olivieri* | FMNH 190620 | KE | Nyeri | 95 | -0.33 | 36.50 | IV-A | 1 | KF684111 | KF690190 | KF684271 |  |  |  |  |
| *C. olivieri* | UC GAN39 | LIB | Gangra | 96 | 7.56 | -8.63 | I-E | 1 | KF684117 | KF699165 KF699199 | KF684270 | KF757108 | KF757139 | KF757176 | KF757209 |
| *C. olivieri* | ZFMK 2011.73 | LIB | Ghi-Jideh Valley | 97 | 5.66 | -8.19 | I-E | 1 | KF684138 | KF690206 | KF684293 |  |  |  |  |
| *C. olivieri* | UC TOK25 | LIB | Tokadeh | 98 | 7.45 | -8.65 | I-E | 1 | KF684132 | KF699169 KF699203 | KF684227 |  |  |  |  |
| *C. olivieri* | FMNH 180966 | MA | Chisongeli Forest | 99 | -16.00 | 35.50 | VIII | 1 | KF684130 | KF690201 | KF684282 |  |  |  |  |
| *C. olivieri* | FMNH 192190 | MA | Dedza Mountain | 100 | -14.25 | 34.33 | IV-A | 1 | KF684144 | KF690209 | KF684277 |  |  |  |  |
| *C. olivieri* | FMNH 191561 | MA | Rumphi | 101 | -10.75 | 33.50 | IV-A | 1 | KF684139 | KF690207 | KF684280 |  |  |  |  |
| *C. olivieri* | CBGP M4920 | MAL | Bamako | 102 | 12.63 | -8.00 | I-C | 1 | KF684104 | KF690183 | KF684273 |  |  |  |  |
| *C. olivieri* | CBGP BAM10 |  |  |  |  |  | II-A | 1 | KF684168 | KF690229 |  |  |  |  |  |
| *C. olivieri* | FMNH 177206 | MOZ | Gurue | 106 | -15.50 | 37.00 | VIII | 2 | KF684150 | KF690214 | KF684292 | KF757120 | KF757148 | KF757188 | KF757219 |
| *C. olivieri* | FMNH 177207 |  |  |  |  |  |  |  | KF684149 | KF690213 | KF684291 | KF757119 | KF757147 | KF757187 | KF757217 |
| *C. olivieri* | FMNH JCK6891 | MOZ | Sofala | 107 | -18.48 | 34.04 | IV-A | 2 | KF684181 |  |  |  |  |  |  |
| *C. olivieri* | CBGP DIV104 | NI | Niamey | 108 | 13.50 | 2.00 | II-Sgl | 5 | JX312017 | KF690193 | JX312055 | KF757115 | KF757145 | KF757182 | KF757214 |
| *C. olivieri* | ZFMK 1988.28 |  |  |  |  |  | I-C | 1 | KF684191 |  |  |  |  |  |  |
| *C. olivieri* | KSU IFE01 | NIG | Ile-Ife | 109 | 7.47 | 4.57 | II-B | 14 | KF684163 | KF690224 |  |  |  |  |  |
| *C. olivieri* | FMNH 207291 | RW | Gasare | 110 | -2.45 | 29.15 | IV-A | 3 | KF684146 | KF690211 | KF684279 |  |  |  |  |
| *C. olivieri* | FMNH 207299 |  |  |  |  |  |  |  | KF684145 | KF690210 | KF684278 |  |  |  |  |
| *C. olivieri* | IVB NK1081 | SE | Wassadou | 113 | 13.35 | -13.34 | I-F | 1 | KF684097 | KF690176 | KF684252 | KF757111 | KF757142 | KF757178 | KF757212 |
| *C. olivieri* | SMNS 31538 | SU | Gemmeiza | 114 | 7.00 | 29.00 | IV-B | 3 | KF684171 | KF690231 |  |  |  |  |  |
| *C. olivieri* | FMNH 147363 | TZ | Ambangulu | 115 | -5.00 | 38.50 | IV-A | 3 | KF684125 | KF690198 | KF684266 |  |  |  |  |
| *C. olivieri* | FMNH 155373 | TZ | Chita | 116 | -8.50 | 35.85 | IV-A | 3 | KF684091 | KF690170 | KF684254 |  |  |  |  |
| *C. olivieri* | FMNH 155376 |  |  |  |  |  |  |  | KF684090 | KF690169 | KF684251 |  |  |  |  |
| *C. olivieri* | FMNH 177840 | TZ | Kigoma | 117 | -4.85 | 29.70 | IV-A | 1 | KF684092 | KF690171 | KF684255 |  |  |  |  |
| *C. olivieri* | FMNH 166701 | TZ | Kilosa | 118 | -6.85 | 37.00 | IV-A | 1 | KF684109 | KF690189 | KF684264 |  |  |  |  |
| *C. olivieri* | FMNH 161221 | TZ | Mhonda | 119 | -6.15 | 37.50 | IV-A | 1 | KF684154 | KF690217 | KF684284 |  |  |  |  |
| *C. olivieri* | FMNH 192912 | TZ | Misenye | 120 | -1.10 | 31.50 | IV-A | 1 | KF684086 | KF690166 | KF684261 |  |  |  |  |
| *C. olivieri* | FMNH 174116 | TZ | Moshi | 121 | -3.33 | 37.33 | IV-A | 2 | KF684128 | KF690199 | KF684269 | KF757114 | KF757144 | KF757181 | KF757213 |
| *C. olivieri* | FMNH 192512 |  |  |  |  |  |  |  | KF684088 | KF690168 | KF684260 |  |  |  |  |
| *C. olivieri* | FMNH 171295 | TZ | Sumbawanga | 122 | -8.00 | 31.60 | IV-A | 1 | KF684115 | KF690191 | KF684265 |  |  |  |  |
| *C. olivieri* | IZEA 2353 | TO | Lome | 123 | 6.13 | 1.22 | II-B | 4 |  | DQ305256 |  |  |  |  |  |
| *C. olivieri* | FMNH 157408 | UG | Bufumbira | 124 | -1.16 | 29.85 | IV-A | 5 | KF684107 | KF690188 | KF684272 |  |  |  |  |
| *C. olivieri* | FMNH 160134 |  |  |  |  |  |  |  | KF684158 | KF690220 | KF684285 |  |  |  |  |
| *C. olivieri* | FMNH 160148 |  |  |  |  |  |  |  | KF684155 | KF690218 | KF684289 |  |  |  |  |
| *C. olivieri* | FMNH 144191 | UG | Kasese | 125 | 0.15 | 30.08 | IV-A | 1 | EF507189 | KF690187 | KF684267 |  |  |  |  |
| *C. olivieri* | FMNH 165091 | UG | Masindi | 126 | 1.60 | 31.70 | IV-A | 1 | KF684136 | KF690204 | KF684275 |  |  |  |  |
| *C. olivieri* | IVB RS1544 | ZA | Ntumbachushi Falls | 127 | -9.85 | 28.94 | IV-C | 1 | KF684085 | KF690165 | KF684258 |  |  |  |  |
| *C. somalica* | IVB ET281 | ETH | Mago | 129 | 5.67 | 36.42 | VII | 2 |  | KM658986 | KM658993 |  |  |  |  |
| *C. somalica* | IVB ET282 |  |  |  |  |  |  |  | KM658983 | KM658987 | KM658994 |  |  |  |  |
| *C. viaria* | CBGP M4644 | MAL | Gatié-Djirma | 104 | 15.73 | -4.82 | III | 1 | KF684179 |  |  |  |  |  |  |
| *C. viaria* | CBGP M4812 | MAL | Sare Mama | 105 | 14.89 | -4.04 | III | 2 | KF684182 |  |  |  |  |  |  |
| *C. viaria* | IZEA 2616 | MO | Massa | 106 | 29.67 | -9.67 | III | 5 | KF684167 | DQ305265 |  |  |  |  |  |
| *C. viaria* | CBGP DIV100 | NI | Niamey | 103 | 13.50 | 2.00 | III | 4 | KF684119 | KF690195 | KF684216 | KF757131 | KF757157 |  | KF757226 |
| *C. viaria* | MNHN 1981-778 | SE | Bandia | 112 | 14.62 | -17.02 | III | 3 | KF684188 |  | KF684295 |  |  |  |  |
| *C. viaria* | MNHN 1992-538 | SE | Richard-Toll | 113 | 16.45 | -15.67 | III | 2 | KF684187 |  | KF684288 |  |  |  |  |
| *C. flavescens* | FMNH GB40787 | SA | Mpumalanga |  |  |  |  |  | EF524822 | EF524717 EF524608 |  | EF525079 |  |  |  |
| *C. flavescens* | IZEA 2582 | SA | Durban |  |  |  |  |  |  | DQ305259 |  |  |  |  |  |
| *C. hirta* | MNHN KP159 | TZ | Ngarembe |  |  |  |  |  | EF524875 | EF524766 EF524660 |  | EF525133 |  |  |  |
| *C. hirta* | MNHN KP106 | TZ | Ngarembe |  |  |  |  |  | EF524877 | EF524768 EF524661 |  | EF525135 |  |  |  |
| *C. hirta* | MNHN KP122 | TZ | Ngarembe |  |  |  |  |  | EF524871 | EF524763 EF524656 |  | EF525129 |  |  |  |
| *C. hirta* | FMNH GB40208 | SA | Limpopo |  |  |  |  |  | EF524821 | EF524716 EF524607 |  | EF525078 |  |  |  |
| *C. lamottei* | SBP BE31 | BE | Wari-Maro Forest |  |  |  |  |  | EF524898 | EF524788 EF524682 |  | EF525156 |  |  |  |
| *C. lamottei* | IZEA 3119 | BF | Nazinga |  |  |  |  |  | EF524807 | EF524593 EF524703 |  | EF525063 |  |  |  |
| *C. buettikoferi* | MNHN 2012-1052 | GU | Mount Nimba |  |  |  |  |  | JQ732595 | JQ732368 KF690150 | JQ732746 | JQ732490 |  |  |  |
| *C. buettikoferi* | MNHN 2012-1066 | GU | Mount Nimba |  |  |  |  |  | JQ732695 | JQ732758 KF690149 | JQ732732 | JQ732554 |  |  |  |
| *C. obscurior* | MNHN 2012-1486 | CI | Azagny |  |  |  |  |  | KC683974 | KC684094 | KC684040 | KC684162 | KC684232 | KC684196 | KC684217 |
| *C. obscurior* | MNHN 2012-1493 | CI | Lamto |  |  |  |  |  | KC683982 | KC684102 | KC684045 | KC684168 | KC684235 | KC684197 | KC684218 |
| *C. suaveolens* | IZEA 4200 | RU | Stravopol |  |  |  |  |  | EF524817 | EF524712 EF524603 |  | EF525074 |  |  |  |
| *C. suaveolens* | IZEA 7508 | CHI | Turpan Xinjiang |  |  |  |  |  |  | EF524700 EF524590 |  | EF525060 |  |  |  |
| *C. nimbae* | SBP R24302 | CI | Tai National Park |  |  |  |  |  | EF524811 | EF524597 |  | EF525067 |  |  |  |
| *Suncus murinus* | IZEA V554 | IN | Masinagudi |  |  |  |  |  | EF524885 | EF524777 EF524669 |  | EF525144 |  |  |  |
| *Suncus murinus* | FMNH LRH 3544 | PHI | Negros Island |  |  |  |  |  | EF524864 | EF524756 |  | EF525122 |  |  |  |
| *Suncus megalura* | MNHN 2012-1191 | GU | Mount Nimba |  |  |  |  |  | JQ732624 | JQ732391 | JQ732273 | JQ732520 |  |  |  |
| *Suncus megalura* | MNHN 2012-1194 | GU | Mount Nimba |  |  |  |  |  | JQ732649 | JQ732413 | JQ732297 | JQ732519 |  |  |  |
