# Supplementary material for: Phylogeography and evolutionary history of the Crocidura olivieri complex (Mammalia, Soricomorpha): from a forest origin to broad ecological expansion across Africa
Source: BMC Evol Biol. 2015 Apr 23;15:71. doi: 10.1186/s12862-015-0344-y (PMC4422046; doi:10.1186/s12862-015-0344-y)
Supplement: Additional file 7: — Markers used in this study. Markers used in this study with specific primers designed to amplify cytb and COI markers. [file 12862_2015_344_MOESM7_ESM.doc]

**Additional file 7**

| Marker | Forward primer | Reverse primer | Length (bp) |
| --- | --- | --- | --- |
| 16S | Ar  (Palumbi *et al*., 1991) | and Hm  (Quérouil *et al*., 2001) | 535 |
| Cytochrome *b* | L14724  (Kocher *et al*., 1989) | H15915  (Ducroz *et al*., 2001) | 1166 |
| Cytochrome oxidase I | BatL5310  (Robins *et al*., 2007) | R6036R  (Robins *et al*., 2007) | 702 |
|  | COIcroc1F  5’-TTCATTACTCGCTGACTCTTTTC-3’ | COIcroc1R  5’-AGRTGTTGRTAAAGAATRGGGTC-3’ | 652 |
| Breast Cancer 1 | BRCA1f  (Dubey *et al*., 2006) | BRCA1r  (Dubey *et al*., 2006) | 888 |
| Signal Transducer and Activator of Transcription 5A | STATa  (Matthee *et al*., 2001) | STATb  (Matthee *et al*., 2001) | 598 |
| Histone Deacetylase 2 | HDAC2-EX10U (Hassanin *et al*., 2013) | HDAC2-EX11L (Hassanin *et al*., 2013) | 617 |
| RIO Kinase 3 | RIOK3-EX6U  (Hassanin *et al*., 2013) | RIOK3-EX7L  (Hassanin *et al*., 2013) | 799 |

**References**

Dubey S, Zaitsev M, Cosson JF, Abdukadier A, Vogel P. 2006. Pliocene and Pleistocene diversification and multiple refugia in a Eurasian shrew (*Crocidura suaveolens* group). *Molecular Phylogenetics and Evolution* 38:635–647.

Ducroz JF, Volobouev V, Granjon L. 2001. An assessment of the systematics of arvicanthine rodents using mitochondrial DNA sequences: evolutionary and biogeographical implications. *Journal of Mammalian Evolution* 8:173–206.

Hassanin, A., An, J., Ropiquet, A., Nguyen, T. T. & Couloux, A. (2013). Combining multiple autosomal introns for studying shallow phylogeny and taxonomy of mammals: Application to the tribe Bovini (Cetartiodactyla, Bovidae). *Molecular Phylogenetics and Evolution*, 66, 766-775.

Kocher TD, Thomas WK, Meyer A, Edwards SV, Paabo S, Villablanca FX, Wilson AC. 1989. Dynamics of mitochondrial DNA evolution in mammals: amplification and sequencing with conserved primers. *Proceedings of the National Academy of Sciences of the United States of America* 86:6196-6200.

Matthee CA, Burzlaft JD, Taylor JF, Davis SK. 2001. Mining the mammalian genome for artiodactyl systematics. *Systematic Biology* 50:367–390.

Palumbi SR, Martin AP, Romano SL, McMillan WO, Stice L, Grabowski G. 1991. The Simple Fool’s Guide to PCR. Department of Zoology, University of Hawaii, Honolulu, USA.

Quérouil S, Hutterer R, Barrière P, Colyn M, Kerbis Peterhans JC, Verheyen E. 2001. Phylogeny and evolution of reference shrews (Mammalia: Soricidae) inferred from 16s rRNA sequences. *Molecular* *Phylogenetics and Evolution* 20:185–95.

Robins JH, Hingston M, Matisoo-Smith E, Ross HA. 2007. Identifying *Rattus* species using mitochondrial DNA. *Molecular Ecology Notes* 7:717–729.
